# Correlation of the intestinal flora and its metabolites with the colonic transport function in functional constipation

**DOI:** 10.3389/fmicb.2025.1591697

**Published:** 2025-05-21

**Authors:** Lunan Hu, Qihong Liu, Xiao Ke, Peilin Zhao, Wenyi Fang, Yan Ren

**Affiliations:** Department of Gastroenterology, The Second People's Hospital Affiliated to Fujian University of Traditional Chinese Medicine, Fuzhou, China

**Keywords:** functional constipation, colonic transit time, intestinal flora, short-chain fatty acids, bile acids

## Abstract

**Background:**

Functional constipation (FC) is a clinically frequent intestinal disorder. A growing body of evidence emphasizes the link between intestinal microecological imbalance and constipation. However, the microbiota composition associated with FC and the mechanisms by which metabolites influence gut motility remain poorly understood.

**Methods:**

Stool samples were collected from 60 participants (20 FC patients with delayed colonic transit time, 20 FC patients with normal colonic transit time, and 20 healthy controls), and macrogenomics and metabolomics were used to assess the differences in the microbiota and metabolite composition of different colonic transit functions in FC. In addition to assessing clinical symptoms, this study aimed to better understand how intestinal flora contributed to impaired gut motility in FC patients.

**Results:**

Significant microbiota taxonomic differences were observed across different gut dynamics in FC; *Alistipes*, *Akkermansia*, *Oscillibacter*, *Ruthenibacterium*, *Alistipes_onderdonkii*, and *Ruthenibacterium_lactatiformans* were key bacteria in FC patients with delayed colonic transit time; *Roseburia* and *Klebsiella_pneumoniae* were key bacteria in FC patients with normal colonic transit time; and *Escherichia*, *Enterobacter*, *Escherichia_coli*, *Ruminococcus gnavus*, *Enterobacter_cloacae_complex*, and *Megamonas_funiformis* were the key organisms in healthy controls. The metabolomics analysis revealed three differentially abundant short-chain fatty acids: acetic acid, propionic acid, and butyric acid. Furthermore, there were 11 differentially abundant bile acids, including *β*-muricholic acid and nor-deoxycholic acid. Correlation analysis revealed significant correlations between the 14 differential bacteria and the 14 metabolites, Notably, *Roseburia* was positively correlated with butyrate and acetate levels (*FDR* < 0.05). In addition, *Oscillibacter* showed positive correlations with several BAs, including nor-deoxycholic acid, isoallolithocholic acid, *α*-muricholic acid, *β*-muricholic acid, 5α-cholanic acid-3α-ol, and dehydrolithocholic acid (*FDR* < 0.05). The Spearman’s *|r |*value >0.6 combination in the correlation analysis between fecal differential bacteria and differentially abundant metabolites revealed an AUC value of 0.854 between FC patients and healthy controls, indicating good predictive ability.

**Conclusion:**

The identified differences in the composition and metabolites of different colonic transmission-dynamic microbiota in FC further our understanding of the underlying mechanisms involved in FC pathogenesis and may provide new insights into diagnostics and therapeutic interventions.

## Introduction

Functional constipation (FC) is constipation without an organic etiology, with clinical manifestations dominated by defecation difficulty, decreased defecation frequency, or a sense of incomplete defecation that does not meet the diagnostic criteria for constipation-type irritable bowel syndrome (Drossman, 2016; Sperber et al., 2021). Poor diet, living and defecation habits, and psychological factors are associated with an increased risk of developing FC. In recent years, with the prolongation of human lifespan, changes in dietary structure, accelerated pace of life, and changes in psychosocial factors, the FC prevalence rate has increased, with the global prevalence reaching approximately 10.1% (Barberio et al., 2021). Prolonged constipation can significantly reduce patients’ quality of life and even trigger pathologies such as organic lesions of the intestinal tract, cardiovascular and cerebrovascular diseases, and Alzheimer’s disease. Thus, FC presents a substantial potential hazard affecting health. In addition, the pathophysiological mechanism of FC, which primarily involves intestinal dyskinesia, abnormal pelvic floor muscle coordination function, and abnormal rectal sensitivity, remains unclear (Aziz et al., 2020). Based on the different pathophysiological mechanisms, Rome IV further classifies FC into slow-transmission constipation, defecation-impaired constipation, and normal-transmission constipation. Of these, slow-transmission constipation is primarily caused by intestinal dyskinesia, and it clinically manifests via symptoms such as infrequent bowel movements and dry stools. Meanwhile, defecation-impaired constipation is mainly caused by pelvic floor muscle coordination dysfunction, and it clinically manifests in the form of symptoms such as straining and difficulty in defecation. Normal-transit constipation is mainly caused by abnormal rectal sensitivity and compliance, and clinically it mainly manifests as abnormal sensations during bowel movements. Both defecation-disordered constipation and normal-transit constipation are associated with normal colon transit function, but with abnormal defecation function. Targeted intervention drugs for clinical use are lacking; the existing medicine is often laxative drugs, and their therapeutic effect is unsatisfactory (Basilisco, 2020). Furthermore, the frequent use of laxative drugs leads to drug dependence and laxative colonization. Therefore, further research on the mechanisms of FC is necessary to improve the effectiveness of therapeutic strategies.

A growing body of research suggests that disturbances in the intestinal flora play an important role in the pathogenesis of FC, and that these disturbances are particularly associated with intestinal dysbiosis. Although the mechanisms by which the gut flora causes intestinal dyskinesia in FC are unknown, researchers have reached the consensus that restoring the balance of the gut flora may be beneficial for people with FC (Wu et al., 2023). Existing studies have focused on describing the differences in the gut flora between FC patients and healthy individuals, e.g., the gut flora of the FC population is significantly different from that of the healthy population (Mancabelli et al., 2017; Parthasarathy et al., 2016; Wang et al., 2023). In addition, the intestinal flora can further modulate intestinal motility by influencing intestinal flora metabolites, such as short-chain fatty acids (SCFAs) (Martin-Gallausiaux et al., 2021; Pan et al., 2022; Zhuang et al., 2019) and bile acids (BAs) metabolism (Fan et al., 2022; Li Y. Q. et al.,2023; Nakajima et al., 2022; Zhang S. et al., 2021). However, few studies have characterized the gut flora and associated metabolites in FC patients with different colonic transport dynamics. Therefore, exploring the link between the intestinal flora and its metabolites and colonic transport function in FC may provide new insights into the etiology and pathophysiology of this disease.

In this study, we classified FC into delayed colonic transit time (DCTT) and normal colonic transit time (NCTT) according to the pathomechanism of FC-related intestinal dyskinesia via the colonic transit test and identified a novel association between the intestinal microecology and the intestine. Furthermore, we revealed a new association between intestinal microecology and dynamics. To explore the potential mechanistic links between gut microecology and the host, we integrated macrogenomic sequencing and metabolomics of the gut flora to reveal the differences in the structure of the gut flora and their SCFA and BA metabolites in FC patients with different colonic transit dynamics and explored the interactions between different gut flora and metabolites to provide a basis for future studies on the mechanism and treatment of FC.

## Methods

### Participant recruitment

Forty patients with FC diagnosed in outpatient clinics or wards of the Second People’s Hospital affiliated with Fujian University of Traditional Chinese Medicine between January 2022 and January 2023 were collected and divided into the DCTT and NCTT groups (20 per group) according to the colonic transport test results. All participants were evaluated by a physician. Sex was not limited across the groups. The inclusion criteria for the DCTT group were as follows: (1) Met the diagnostic criteria for FC in Rome IV; (2) The colon transport test suggested prolonged colon transport time; (3) Age ranging from 18 to 70 years; BMI ranging from 18.5–23.9; long-term residence in Fujian (more than 10 years); and no smoking or alcohol addiction; (4) No special dietary habits, such as a low-FODMAP diet; a low-fiber diet; a vegetarian diet; or other special diets (diets were classified as low-fiber, vegetarian, meat eater, religious, and other dietary habits); and (5) Voluntary participation and signing of informed consent documentation. The inclusion criteria for the NCTT group were as follows: (1) Met the diagnostic criteria for FC in Rome IV; (2) Had a colonic transmission test suggesting a normal colonic transmission time; (3) Age ranging from 18 to 70 years, BMI ranging from 18.5–23.9, long-term residence Fujian (more than 10 years), and no tobacco or alcohol addiction. The exclusion criteria were as follows: (1) constipation-type irritable bowel syndrome, severe primary or secondary diseases of the heart, brain, liver, kidney, gallbladder, or bacterial overgrowth in the small intestine; (2) Organic intestinal pathology confirmed by electronic colonoscopy; (3) A history of surgery on the gastrointestinal tract, gallbladder, liver, or cranial or cerebral tract; (4) The use of drugs such as antibiotics, microecological agents (probiotics, prebiotics, and synbiotics), and acid inhibitors in the past 3 months; (5) Pregnant or lactating women; and (6) Those with communication or cognitive difficulties. In addition, 20 healthy controls (HCs) were recruited during the same period. The inclusion criteria were as follows: (1) No constipation or diarrhea; (2) Age ranging from 18 to 70 years, Han nationality, BMI ranging from 18.5–23.9, long-term residence in Fujian (more than 10 years), no tobacco or alcohol addiction; (3) No use of antibiotics in the last 3 months or drug such as those causing constipation and diarrhea, microecological preparations (probiotics, probiotics, synbiotics), and acid inhibitors; (4) the absence of organic intestinal pathology, pregnancy or lactation, and history of intestinal surgery or drug addiction; (5) The absence of other cardiocerebral or cerebral vascular, hepatic, renal, or endocrine metabolism, inflammatory bowel, malignant tumor, or other diseases; and (6) Voluntary participation and signing of informed consent documentation. The study was approved by the Ethical Review Committee of the Second People’s Hospital affiliated with Fujian University of Traditional Chinese Medicine (SPHFJP-Y2023053-01), and all participants provided written informed consent.

### Data acquisition

A colonic transmission test was used to determine the time of FC colonic transmission: 7 days before the examination, colonoscopy was prohibited, and drugs that affect gastrointestinal dynamics, promote defecation, or stimulate food were stopped. Corkscrew use was stopped 3 days before the examination. During the examination period, corkscrews and laxatives to assist in defecation were prohibited, as were laxative foods, such as bananas and honey. The patients recorded the number of bowel movements. Standard meal-impermeable X-ray markers (1 mm in diameter, 10 mm in diameter, 20 markers) were employed; at 48 h, if the number of markers present above the sigmoid colon vs. the number of total markers exceeded 70%, an X-ray was taken to determine the presence of delayed colonic transmission. The marker excretion rate was calculated as follows: rate = (total number of markers – number of marker residues)/total number of markers × 100%. A marker excretion rate ≥ 80% at 72 h indicated a normal colon transit time; a value < 80% indicated a delayed colon transit time. The severity of constipation in FC patients was assessed via the Chronic Constipation Severity Score (CSS). The Bristol Stool Scale (BSFS) was used to score the participants’ feces (Chumpitazi et al., 2016), and the number of spontaneous bowel movements (SBMs) per week was recorded.

### Fecal sample collection and transportation

A total of 6 g of fresh fecal sample from each group was divided into three sterile freezing tubes, quickly stored at −80°C for 5 min, and subsequently transported on dry ice for examination.

### Analysis of the fecal intestinal flora

DNA extraction, library construction, and macrogenomic sequencing: Fecal DNA was extracted using the ALFA-SEQ Advanced Stool DNA Kit per the manufacturer’s instructions. Fecal DNA concentration and purity were determined via a Qubit 4.0 instrument (Thermo Fisher Scientific, Waltham, MA, United States) and a NanoDrop One instrument (Thermo Fisher Scientific, Waltham, MA, United States) to determine the DNA concentration and purity. Qualified DNA samples were added to fragmentation buffer and randomly disrupted via an ultrasonic crusher, and the short fragments of DNA obtained after interruption were used for library construction. A Qsep400 high-throughput nucleic acid protein analysis system was used to evaluate the size of the library fragments, a Qubit 4.0 was used to measure the concentration of the library, and the libraries that passed the quality inspection were sequenced using the Illumina HiSeq 2,500 high-throughput sequencing platform for PE150 sequencing.

Data analysis: (1) The raw sequencing data were processed using Trimmomatic with the following parameters: LEADING:3, TRAILING:3, SLIDINGWINDOW:5:20, and MINLEN:50. After quality control, clean reads were obtained. The quality control procedure included the following steps:① LEADING:3—removal of bases from the start of a read if the base quality was below 3; ② TRAILING:3—removal of bases from the end of a read if the base quality was below 3; ③ SLIDINGWINDOW:5:20—application of a sliding window of five bases from the 5′ end, trimming the read when the average base quality within the window dropped below 20; ④ MINLEN:50—discarding reads shorter than 50 bases after trimming; ⑤ Removal of adapter sequences; ⑥ Filtered reads were aligned to the host genome using Bowtie2 to remove potential host-derived DNA contamination. Clean data were *de novo* spliced via MEGAHI after QC, and the reads not utilized for each sample were combined for mixed assembly. After splicing, the scaffolds obtained from the assembly were broken from the N junction to obtain the N-free sequence fragment scaftig, and the fragments larger than 500 bp in all scaftigs were screened for subsequent statistical analysis. (2) Data statistics and visualization: the screened scaftigs were used to predict open reading frames (ORFs) via MetaGeneMark, a non-redundant gene catalog (unigenes) was obtained via gene clustering, and the redundancy was determined via Linclust software. The unigene sequences of the non-redundant gene set were compared with those of the NCBI-NR database for species annotation, and the species annotation information of the unigenes was obtained. The data were combined with the gene abundance table, and species composition and abundance data were obtained for each taxonomic tier. Furthermore, species diversity, composition, and differentiation analyses were performed. The predicted protein sequences of the genes were compared with the Kyoto Encyclopedia of Genes and Genomes (KEGG) database to obtain functional annotation information. Macrogenome sequencing was completed by Guangdong MegaGene Technology Co.

### Analysis of fecal SCFA levels

Chemicals and reagents: Methyl tert-butyl ether (MTBE) was purchased from CNW (CNW Technologies, Germany). Milli-Q water (Millipore, Bradford, USA) was used in all the experiments. All standards were purchased from CNW (Beijing) or Aladdin (Shanghai). The standard stock solutions were prepared at a concentration of 1 mg/mL in methyl tert-butyl ether (MTBE). All stock solutions were stored at −20°C. The stock solutions were diluted with MTBE to yield working solutions before analysis.

Sample preparation and extraction: A 20 mg fecal sample was accurately weighed and placed in a 2 mL EP tube. Next, 1 mL of phosphoric acid (0.5% v/v) solution and a small steel ball were added to the EP tube. The samples were ground uniformly, vortexed for 10 min, and ultrasonicated for 5 min. Then, 100 μL of the supernatant was transferred to a 1.5 mL EP tube. After the mixture was centrifuged at a centrifugal force of 11,304 × g for 10 min at 4°C, it was transferred to a 1.5 mL centrifuge tube. Subsequently, 500 μL of MTBE (containing an internal standard) solution was added to the centrifuge tube. Standard solution was added to the centrifuge tube, and the mixture was vortexed for 3 min, followed by ultrasonication for 5 min. The mixture was then centrifuged twice at a centrifugal force of 11,304 × g for 10 min at 4°C. The supernatant was collected and used for GC–MS/MS analysis.

GC–MS analysis: an Agilent 7890B gas chromatograph coupled to a 7000D mass spectrometer with a DB-FFAP column (30 m length × 0.25 mm i.d. × 0.25 μm film thickness, J&W Scientific, USA) was used for GC–MS/MS analysis of SCFAs. Helium was used as the carrier gas at a flow rate of 1.2 mL/min. Injection was performed in split mode with a split ratio of 5:1, and the injection volume was 1 μL. The oven temperature was held at 50°C for 1 min, increased to 220°C at a rate of 18°C/min, and held for 1 min. The oven temperature was held at 50°C for 1 min, increased to 220°C at a rate of 18°C/min, and held for 5 min. All samples were analyzed in multiple reaction monitoring modes at 250 or 230°C.

### Analysis of fecal BAs

Chemicals and reagents: HPLC-grade acetonitrile (ACN) and methanol (MeOH) were purchased from Merck (Darmstadt, Ger many). Milli-Q water (Millipore, Bradford, USA) was used in all the experiments. All of the standards were purchased from CNW (Shanghai, China) and IsoReag (Shanghai, China). Acetic acid and ammonium acetate were purchased from Sigma–Aldrich (St. Louis, MO, USA). The standard stock solutions were prepared at a concentration of 1 mg/mL in MeOH. All stock solutions were stored at −20°C and diluted with MeOH to yield working solutions before analysis.

Sample preparation and extraction: Samples (20 mg) were extracted with 500 μL of methanol after grinding in a ball mill. A 10 μL internal standard mixture (1 μg/mL) was added to the extract as an internal standard for quantification. The samples were incubated at −20°C for 10 min to precipitate the protein. Then, the samples were centrifuged for 10 min (with a centrifugal force of 11,304 × g and at 4°C). After centrifugation, the supernatant was passed through a protein precipitation plate for further LC–MS analysis.

HPLC conditions: the sample extracts were analyzed via an LC–ESI–MS/MS system (UHPLC ExionLC™ AD https://sciex.com.cn/; MS Applied Biosystems 6,500 Triple Quadrupole, https://sciex.com.cn/). The analytical conditions were as follows. HPLC: column, Waters ACQUITY UPLC HSS T3 C18 (100 × 2.1 mm i.d. 1.8 μm); solvent system, water with 0.01% acetic acid and 5 mmol/L acetic acid and 5 mmol/L ammonium acetate (A), acetonitrile with 0.01% acetic acid (B); the gradient was optimized at 5 to 40% B in 0.5 min, then increased to 50% B in 4 min, then increased to 75% B in 3 min, and then 75 to 95% in 2.5 min, washed with 95% B for 2 min, and finally ramped back to 5% B (12–14 min); flow rate, 0.35 mL/min; temperature, 40°C; injection volume: 1 μL. The effluent was alternatively connected to an ESI-triple quadrupole-linear ion trap (QTRAP)-MS.

ESI–MS/MS conditions: Linear ion trap and triple quadrupole scans were acquired on a triple quadrupole–linear ion trap mass spectrometer (QTRAP), a QTRAP^®^ 6,500 + LC–MS/MS system, equipped with an ESI Turbo Ion–Spray interface, operated in negative ion mode and controlled by Analyst 1.6.3 software (Sciex). The ESI source operating parameters were as follows: ion source, ESI-; source temperature, 550°C; ion spray voltage (IS), 4,500 V; and curtain gas (CUR) pressure, 35 psi. Bile acids were analyzed using scheduled multiple reaction monitoring. Data acquisition was performed via Analyst 1.6.3 software (Sciex). Multiquant 3.0.3 software (Sciex) was used to quantify all the metabolites. The mass spectrometer parameters were analyzed, including the declustering potentials and collision energies. Mass spectrometer parameters, including declustering potentials and collision energies, for individual multiple reaction monitoring transitions were determined and further optimized. A specific set of MRM transitions was monitored for each period according to the metabolites eluted within this period.

### Statistical analysis

The data were entered and analyzed via IBM SPSS 24.0 software (IBM Corporation, Armonk, NY, United States) and GraphPad Prism software (version 10). Descriptive data with a normal distribution are expressed as the mean ± standard deviation (± s). Data not conforming to a normal distribution are expressed as the median with quartiles, i.e., M (*P25, P75*). For normally distributed data, one-way ANOVA was used for comparisons between multiple groups, and further two-by-two comparisons were made via the least significant difference (LSD) method when the variance was homogeneous. The Games–Howell method was used when the variance was not homogeneous. A nonparametric rank sum test was used for data that did not satisfy a normal distribution. Comparisons of count data were performed via the chi-square test. Correlations between intestinal flora, intestinal metabolites and constipation symptoms were analyzed using Spearman’s correlation coefficient and corrected *p*-values using the false discovery rate (FDR) method. ROC curves were plotted, and the area under the curve (AUC) was calculated to evaluate the discriminatory accuracy of the differential flora and differentially abundant metabolites to determine whether these biomarkers were diagnostically significant. In all analyses, *p* < 0.05 was considered to indicate significance.

## Results

### Participant characteristics

This study included 20 patients with DCTT, 20 patients with NCTT, and 20 HCs; their demographic and clinical symptomatic data are detailed in Table 1. The differences in age, sex, BMI and duration of disease among the three groups were not significant and were comparable (*p* > 0.05). The number of voluntary bowel movements per week was lower in patients with DCTT than in those with NCTT, as were the BSFS scores (*p* < 0.05).

**Table 1:** Demographic characteristics of study participants.

Variables	DCTT	NCTT	HC	*P*
Age (years)	53.55 ± 17.03	55.40 ± 14.31	52.80 ± 9.85	0.834
Sex (percentage of women)	60%	60%	65%	0.932
Body mass index (BMI)	20.75 ± 1.00	21.12 ± 0.73	20.77 ± 1.01	0.373
Duration of illness (years)	8.85 ± 3.95	7.85 ± 3.25	-	0.387
CSS score (points)	11.85 ± 2.91	11.00 ± 3.95	-	0.443
BSFS score (points)	1.00 (1.00,2.00)	2.00 (1.00,4.00)	4.00 (3.00,4.00)	0.002
Number of SBMs per week (times)	2.00 (2.00,3.00)	4.50 (2.00,6.75)	6.00 (5.00,7.00)	< 0.001

### Intestinal flora analysis results

#### Overview of the gene catalog

After quality control of the sequencing data, a total of 642,940,796,404 base pairs of clean bases and 4,324,539,572 high-quality clean reads were obtained. Quality control statistics for each individual sample are provided in Supplementary Table S1. Subsequent *de novo* assembly resulted in 6,118,534 scaftigs, with a total assembled length of 14,647,589,706 base pairs. Detailed assembly statistics for each sample are shown in Supplementary Table S2.

#### Intestinal flora species composition analysis results

**Figure 1:**
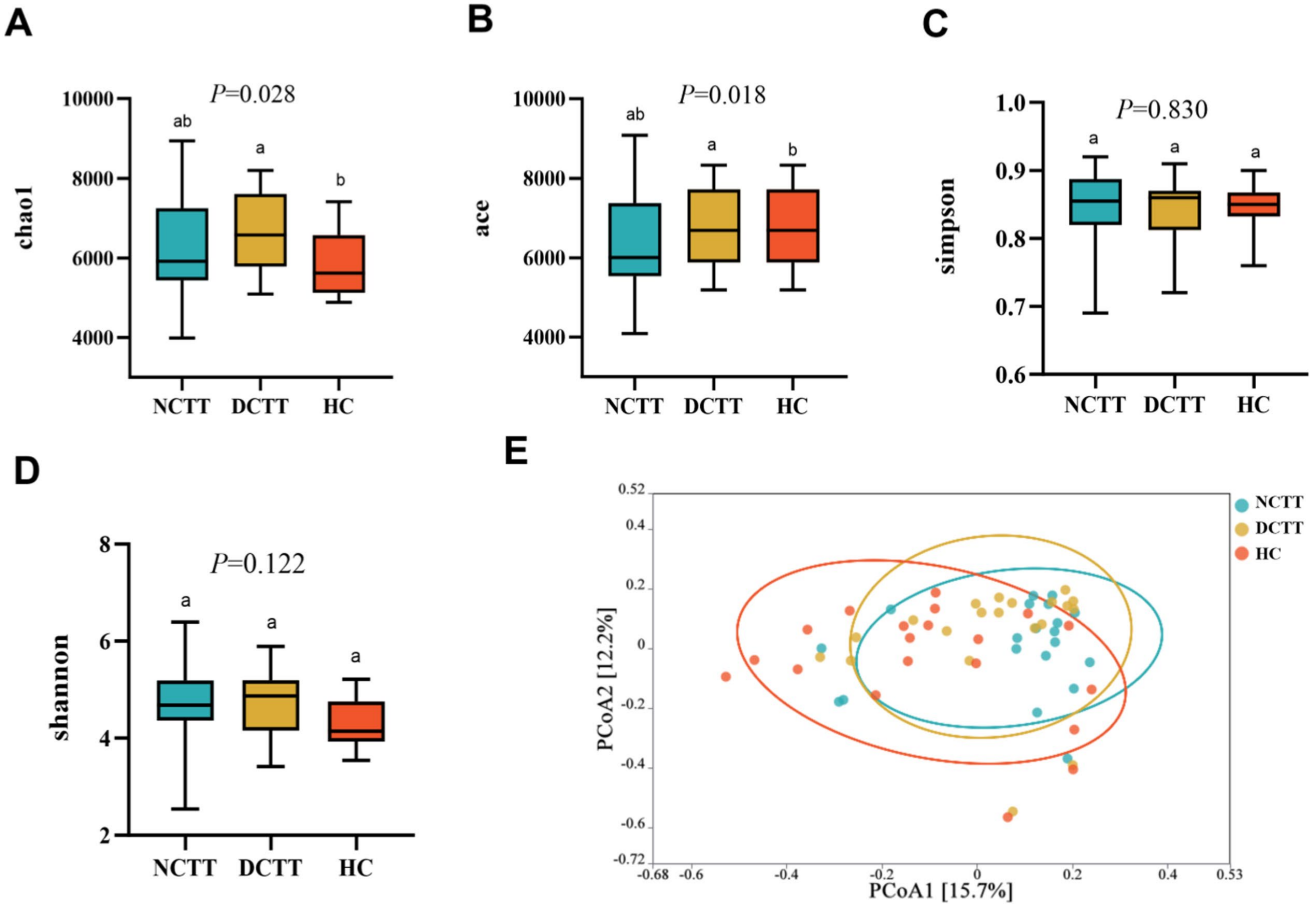
Comparison of the three groups in terms of richness index (**A**) chao1, (**B**) ace, (**C**) Simpson, and (**D**) Shannon. (**E**) Principal coordinate analysis (PCoA) of the three groups of intestinal flora according to Bray–Curtis analyses. NCTT normal colonic transit time, DCTT delayed colonic transit time, HC healthy control participant. In the figures and tables, different letters denote statistically significant differences among groups (*p* < 0.05). Groups that share the same letter are not significantly different from each other.

The alpha diversity analysis revealed that the richness indices ace and chao1 were greater in the DCTT group than in the HC group (*p* < 0.05); the difference from the NCTT group was not significant (*p* > 0.05) (Figures 1A,B). The differences in the Simpson and Shannon diversity indices among the three groups were not significant (*p* > 0.05) (Figures 1C,D). The significance analysis of the differences in community structure between groups revealed that the differences were not significant between the three groups of intestinal flora at the phylum level (Anosim R = 0.0198, *p* = 0.146). However, significant differences in community structure were observed between groups at the genus (Anosim R = 0.0708, *p* = 0.001) and species (Anosim R = 0.0917, *p* = 0.002) levels (see Table 2). At the genus level, the DCTT group differed significantly from the NCTT and HC groups in terms of community structure. The NCTT group had a colony structure similar to that of the HC group, and at the species level, there were differences in colony structure between the groups. pCoA analysis revealed a tendency for aggregation of the samples from the DCTT and NCTT groups at the species level and a tendency for segregation from the samples from the HC group (Figure 1E).

**Table 2:** Results of Anosim analysis of the NCTT, DCTT, and HC groups.

Level	Dist	Test	Group	R-value	*P* value
Phylum	Bray	Anosim	DCTT vs. HC	0.00592	0.322
Phylum	Bray	Anosim	DCTT vs. NCTT	0.0312	0.122
Phylum	Bray	Anosim	HC vs. NCTT	0.0197	0.182
Phylum	Bray	Anosim	All groups	0.0198	0.146
Genus	Bray	Anosim	DCTT vs. HC	0.109	0.006
Genus	Bray	Anosim	DCTT vs. NCTT	0.0556	0.034
Genus	Bray	Anosim	HC vs. NCTT	0.0512	0.054
Genus	Bray	Anosim	All groups	0.0708	0.001
Species	Bray	Anosim	DCTT vs. HC	0.155	0.001
Species	Bray	Anosim	DCTT vs. NCTT	0.0473	0.046
Species	Bray	Anosim	HC vs. NCTT	0.0775	0.019
Species	Bray	Anosim	All groups	0.0917	0.002

The analysis of the significance of differences in community structure between groups was based on the Bray–Curtis distance algorithm to determine whether there was a significant difference in species diversity between different samples. Anosim analysis was used to derive an R-value, with R between (−1, 1), R > 0 indicating that the differences between groups were significant, and *P* < 0.05 suggesting that the differences were statistically significant.

**Figure 2:**
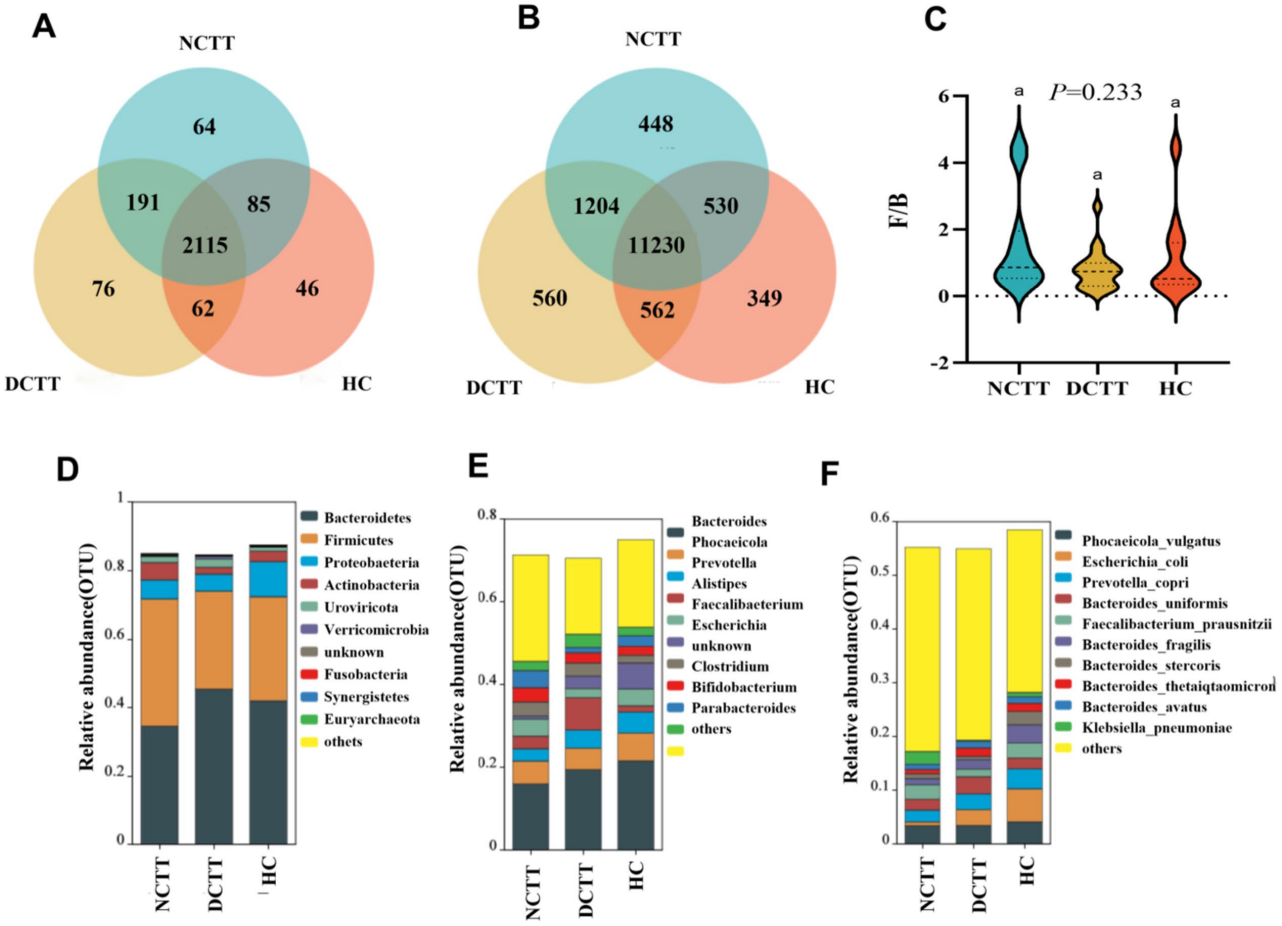
Analysis of shared and endemic composition of groups at the **(A)** genus and **(B)** species level. **(C)** Analysis of the F/B ratio of groups using the Kruskal–Wallis test, with F/B being the ratio of relative abundance of Firmicutes to Bacteroidetes. Relative abundance levels of the top 10 microbial groups in the three groups at the **(D)** phylum, **(E)** genus, and **(F)** species levels.

Species composition was analyzed, revealing a total of 2,115 shared genera and 11,230 shared species between the three groups, shown as a Venn diagram; 76 endemic genera and 560 endemic species overlapped in the DCTT group; and 64 endemic genera and 448 endemic species in the NCTT group (Figures 2A,B). Classification via OTU species annotation revealed that the relative abundance composition of the species in the three groups of intestinal flora was dominated by the phyla thick-walled *bacteria* and *Bacteroidetes* at the phylum level, and the F/B ratio difference among the groups was not significant (*p* > 0.05); however, the F/B ratio in the DCTT group tended to be lower than that in the HC group and the NCTT group (Figures 2C,D). At the genus level, *Bacteroides* and *Phocaeicola* were the dominant genera in the NCTT and HC groups, *Bacteroides* and *Alistipes* were the dominant genera in the DCTT group; the top 10 genera in terms of the relative abundance of species in each group are shown in Figure 2E. At the species level, *Escherichia_coli* was the dominant genus in the HC group, whereas *Phocaeicola_voli* was the dominant genus in the DCTT group, along with *Phocaeicola_vulgatus* and *Bacteroides_uniformis*. The NCTT group was dominated by *Phocaeicola_vulgatus* and *Faecalibacterium_prausnitzii*. The top 10 species ranked by the relative abundance of species in each group are shown in Figure 2F.

**Figure 3:**
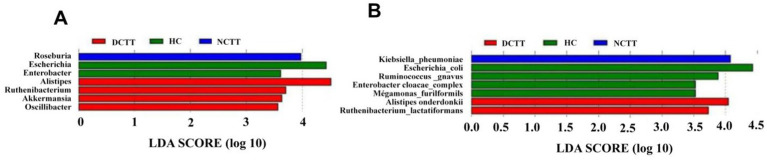
LEfSe analysis of three sets of LDA histograms at the **(A)** genus and **(B)** species level, with LDA score plots showing differential genera. Higher LDA scores indicate a greater contribution of bacterial genera to the variance.

To identify and test potential diagnostic intestinal flora biomarkers, we further applied LEfSe analysis and screened a total of 14 key organisms via linear discriminant analysis (LDA) = 3.5, revealing that *Roseburia* and *Klebsiella_pneumoniae* were key organisms in the NCTT group, namely, *Alistipes*. *Akkermansia*, *Oscillibacter*, *Ruthenibacterium*, *Alistipes_onderdonkii*, and *Ruthenibacterium_lactatiformans* were the key organisms in the DCTT group, whereas *Escherichia, Enterobacter*, *Escherichia_coli*, *Ruminococcus gnavus*, *Enterobacter_cloacae_complex*, and *Megamonas_funiformis* were the key bacteria in the HC group (Figures 3A,B). The relative abundances of the key bacteria in each group are shown in Supplementary Figure S1.

#### Potential functions of the gut microbiome in FC progression

**Figure 4:**
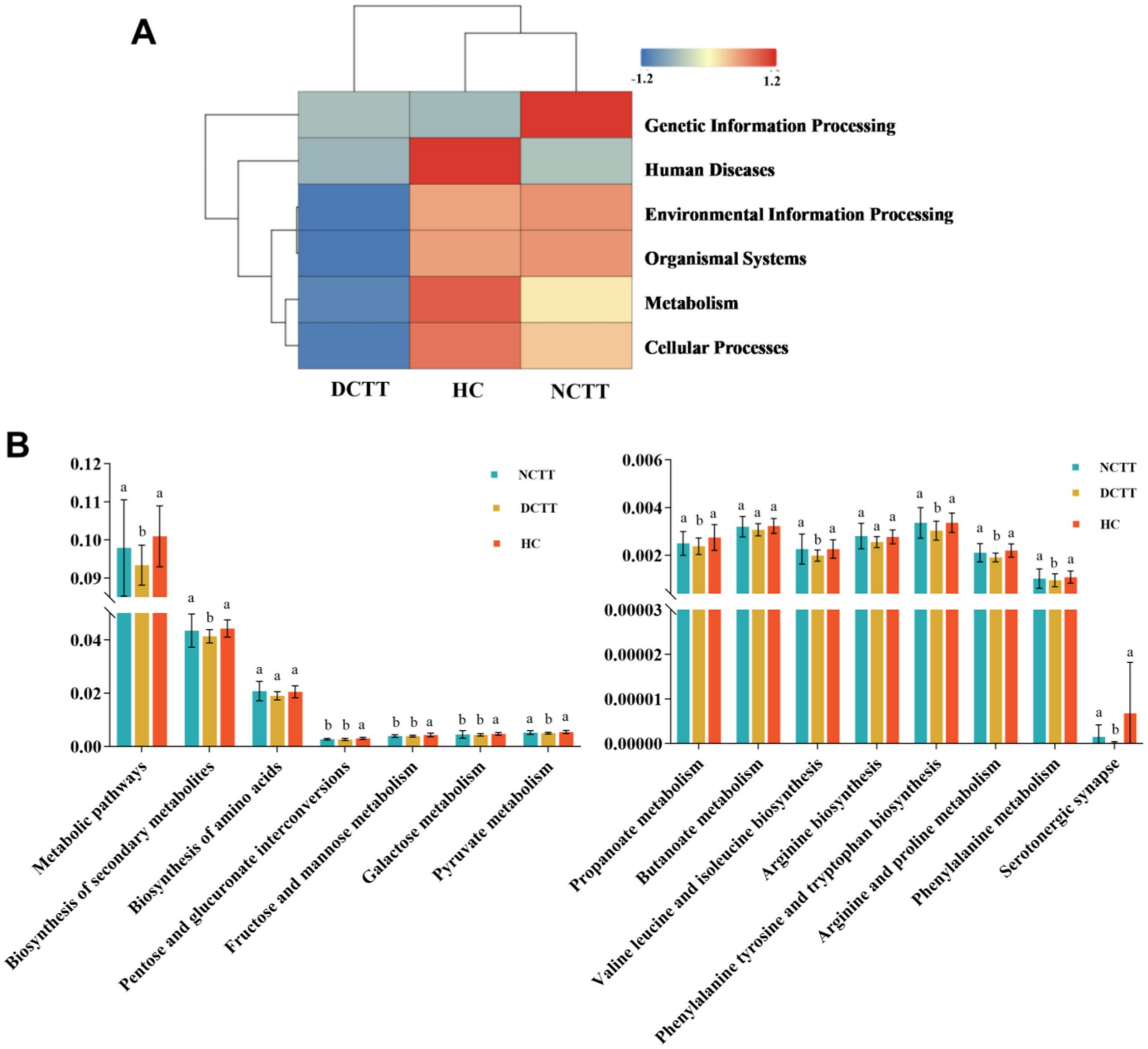
**(A)** Cluster analysis of KEGG functional level abundance. **(B)** Differential analysis of intestinal flora function in each group.

The KEGG database was used to compare sequencing data and obtain the corresponding pathways and functions. A total of six pathway functions were involved in KEGG prediction, with a focus on the metabolism pathway, and the function level of the DCTT group was significantly lower than that of the NCTT and HC groups (*p* = 0.027). Abundance clustering function analysis revealed that the gene functions of the DCTT group clustered differently from those of the NCTT and HC groups, whereas the gene function clustering of the NCTT group was similar to that of the HC group (Figure 4A). The metabolic pathways analysis revealed that the following pathways were significantly lower in the DCTT group than those in the NCTT and HC groups: biosynthesis_of_secondary_metabolites, pyruvate metabolism, propanoate metabolism, valine leucine and isoleucine biosynthesis, arginine and proline metabolism, phenylalanine metabolism, phenylalanine tyrosine and tryptophan biosynthesis, and serotonergic synapse functional level. Pentose and glucuronate interconversions, fructose and mannose metabolism, and galactose metabolism demonstrated lower functional levels in the DCTT and NCTT groups versus the HC group (Figure 4B).

### Fecal metabolite analysis

**Figure 5:**
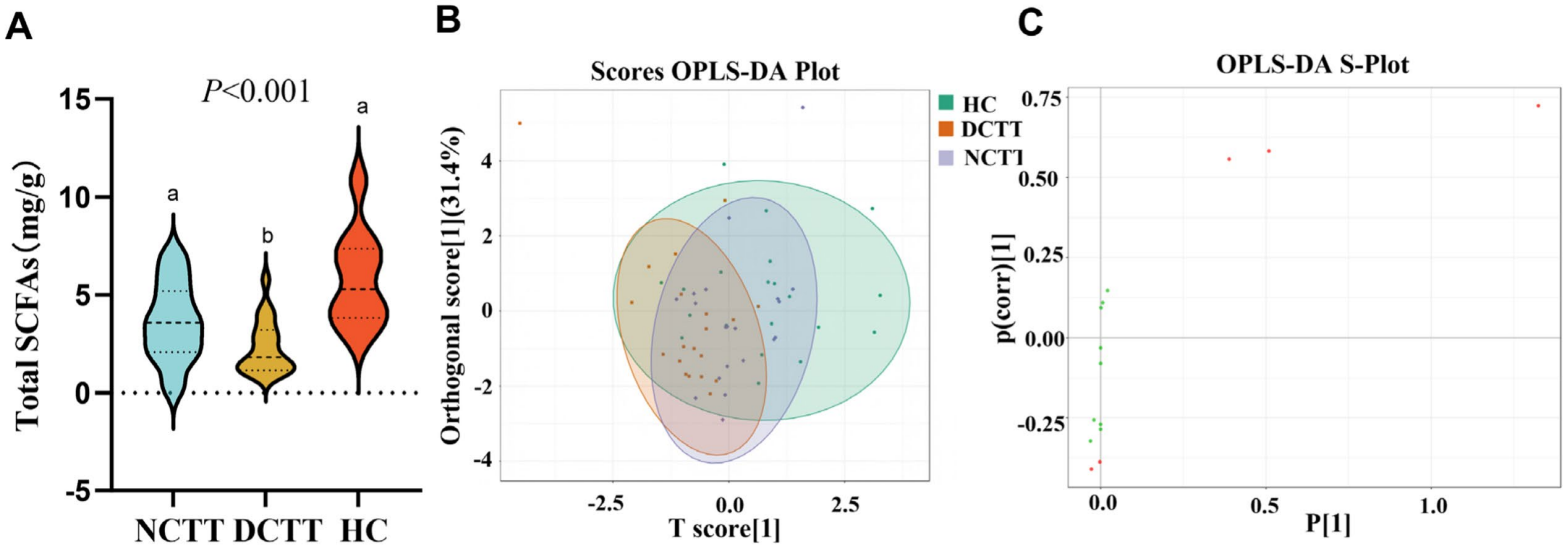
**(A)** Analysis of SCFAs in the three groups. **(B)** Plot of OPLS–DA scores for the three groups, with the horizontal coordinates indicating the predicted principal components. The direction of the horizontal coordinates reveals the intergroup gap. Vertical coordinates indicate orthogonal principal components, and the vertical coordinate direction shows the gap within groups. The score ratio indicates the degree of explanation of the data set by this component. **(C)** OPLS–DA S-plot: the horizontal coordinate indicates the covariance between principal components and metabolites, and the vertical coordinate indicates the correlation coefficient between principal components and metabolites. The closer the metabolites are to the upper right and lower left corners, the more significant the difference is. The red dots indicate that the VI*p* value of these metabolites is greater than 1, and the green dots indicate that the VIp value of these metabolites is less than or equal to one.

In this study, we analyzed the levels of fecal SCFAs in FC patients with different colonic transport dynamics (FCs). A total of nine SCFAs, dominated by acetic acid, propionic acid, and butyric acid, were detected via GC–MS. The total SCFA content was lower in the DCTT group than in the NCTT and HC groups (*p* < 0.05), and the difference between the NCTT and HC groups was not significant (Figure 5A). Orthogonal partial least squares discriminant analysis (OPLS–DA) was performed for all metabolites, and the plotting of OPLS–DA scores with an S-plot revealed that there was a difference in SCFAs between the DCTT and NCTT groups and the HC group (Figures 5B,C). On the basis of the variable importance in projection (VIP) obtained from the OPLS–DA model, the metabolites that differed across subgroups could be initially screened. Differentially abundant metabolites were further screened with VIP > 1 and one-way ANOVA. A total of three selected differential SCFAs were screened, and the acetic acid DCTT group demonstrated lower levels than the NCTT and HC groups (*p* < 0.05). The propionic acid content of the DCTT group was lower than that of the NCTT and HC groups (*p* < 0.05). The difference between the DCTT group and the NCTT group was not significant; however, there was a tendency for the DCTT group to demonstrate lower levels than the NCTT group. The butyric acid content of the DCTT group was lower than that of the HC group (*p* < 0.05) (see Table 3), and the difference between the DCTT and NCTT groups was not significant; however, the butyric acid content of the DCTT group tended to be lower than that of the NCTT group. These findings suggest that reduced levels of propionic acid, acetic acid, and butyric acid may be important for the pathogenesis of intestinal dyskinesia in FC.

**Table 3:** Characteristics of the three significantly differentiated SCFAs.

Name	VIP	P-value	RT (s)	Quantitative analysis (DCTT)	Quantitative analysis (NCTT)	Quantitative analysis (HC)
Propionic acid	1.595	< 0.001	6.504	0.5997 ± 0.38493^#^	0.8678 ± 0.63986^#^	1.4108 ± 0.71258
Butyric acid	1.524	0.015	7.109	0.4831 ± 0.31184^#^	0.7768 ± 0.56539	0.9668 ± 0.60725
Acetic acid	1.988	< 0.001	5.897	1.2160 ± 0.71981^#^*	1.9636 ± 1.08856^#^	3.4259 ± 1.35967

^#^*p* < 0.05 compared to HC group; *p < 0.05 compared to NCTT group.

**Figure 6:**
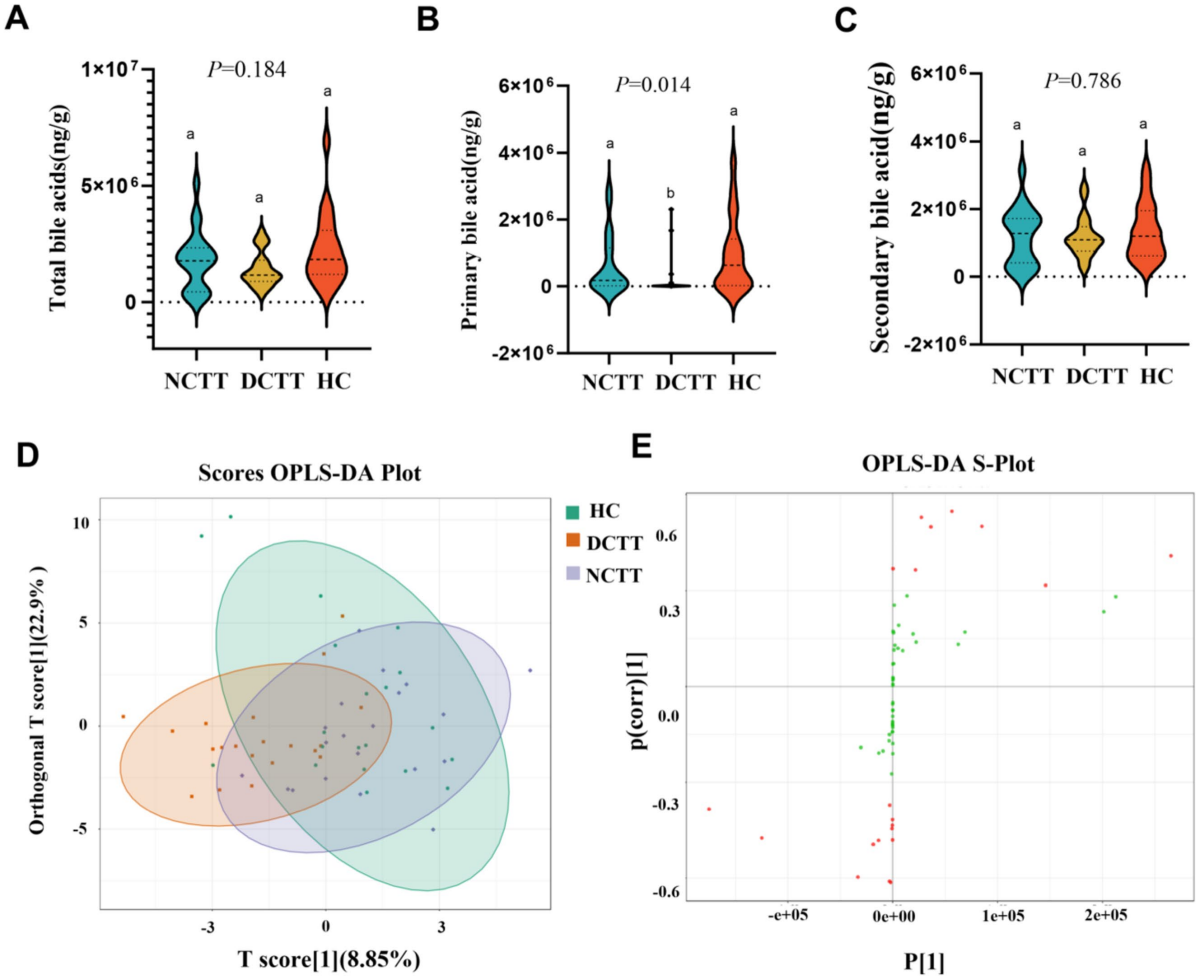
Analysis of variance of **(A)** total bile acids, **(B)** primary bile acids, and **(C)** secondary bile acids across the three groups. **(D)** Plot of OPLS–DA scores of the three groups, with the horizontal coordinate indicating the predicted principal component, and the direction of the horizontal coordinate revealing the gap between the groups. The vertical coordinate indicates the orthogonal principal component, and the direction of the vertical coordinate reveals the gap between the groups. The percentage indicates the component’s interpretation of the data set and the degree to which the component explains the data set. **(E)** OPLS–DA S-plot, the horizontal coordinate indicates the covariance between principal components and metabolites. The vertical coordinate indicates the correlation coefficient between principal components and metabolites. The closer the metabolites are to the upper right and lower left corner, the more significant the difference is. The red dots indicate that the VIp value of these metabolites is greater than 1, and the green dots indicate a VIp value less than or equal to 1.

In this study, we analyzed the fecal BAs in patients with FC with different colonic transport dynamics. A total of 62 BAs were detected via the LC–MS/MS method, in which the difference between the total BA and secondary BA contents of the three groups was not significant, the primary BA content of the DCTT group was significantly lower than that of the HC group (*p* < 0.05), and the difference between the NCTT and HC groups was not significant (Figures 6A–C). OPLS–DA was performed for all metabolites, and the plotting of OPLS–DA scores with an S-plot revealed differences in BAs between the DCTT and NCTT groups and the HC group (Figures 5D,E). Metabolites with different subgroup differences were preliminarily screened out according to the VIP obtained from the OPLS–DA model. Differentially abundant metabolites were further screened by VIP > 1 and the Kruskal–Wallis test, and 11 selected differential BAs were screened (see Table 4). Nor-deoxycholic acid, isoallolithocholic acid, *α*-muricholic acid, *β*-muricholic acid, 5α-CHOLANIC CID-3α-OL, and dehydrolithocholic acid were significantly greater in the DCTT group than in the HC group (*p* < 0.05), and the difference between the NCTT and HC groups was not statistically significant. The levels of isoballolithocholic acid, α-muricholic acid, cholic acid 7-sulfate, and β-muricholic acid in the DCTT group were significantly greater than those in the NCTT group (*p* < 0.05). The levels of ursodeoxycholic acid, chenodeoxycholic acid, 3β-ursodeoxycholic acid, and 7-ketolithocholic acid were significantly lower in the DCTT group than those in the HC group (*p* < 0.05), and the difference between the NCTT and HC groups was not significant. Chenodeoxycholic acid was lower in the DCTT group than that in the NCTT group (*p* < 0.05), suggesting that abnormal metabolism of BAs is a key mechanism in FC-induced intestinal dyskinesia.

**Table 4:** Characterization of 11 significantly differentiated metabolites.

Name	VIP	Q-value	RT (s)	Quantitative analysis (DCTT)	Quantitative analysis (NCTT)	Quantitative analysis (HC)
Nor-deoxycholic acid	1.614	0.042	9.90	188.133 (94.443, 463.745) ^#^	58.431 (1.291, 177.410)	58.431 (1.291, 177.410)
Isoallolithocholic acid	2.005	0.003	11.29	26868.703 (8703.036, 45303.053) ^#*^	1017.180 (41.397, 13034.239)	732.234 (136.436, 3317.865)
α-muricholic acid	2.061	<0.001	5.03	1970.856 (1376.640, 3896.755) ^#*^	682.952 (197.149, 1389.088)	273.624 (0, 1197.251)
Cholic acid 7 sulfate	1.495	0.025	2.50	401.185 (150.710, 755.668) ^*^	116.243 (24.334, 274.782)	178.322 (74.852, 306.093)
β-muricholic acid	2.049	<0.001	0.89	3114.631 (2304.060, 5004.591) ^#*^	303.293 (0, 1756.048)	107.936 (0, 752.157)
5α-cholanic acid-3α-ol,	1.619	0.035	11.79	16166.652 (2642.879, 23733.062) ^#^	4307.215 (398.404, 14847.046)	303.604 (0, 7627.590)
Ursodeoxycholic acid	1.682	0.037	2.27	407.059 (0, 3698.404) ^#^	5644.500 (393.338, 92664.115)	36024.950 (2896.922, 121417.375)
Dehydrolithocholic acid	1.661	0.012	11.81	24413.163 (13757.761, 43563.965) ^#^	8885.782 (2552.558, 30565.585)	6959.699 (539.363, 20691.869)
Chenodeoxycholic acid	1.373	0.029	5.61	1389.722 (755.012, 13128.439) ^#*^	34638.541 (3263.498, 206958.988)	152347.992 (10392.554, 522394.886)
3β-ursodeoxycholic acid	1.777	0.017	8.25	523.605 (125.861, 1880.908) ^#^	2093.946 (378.080, 33542.240)	11394.847 (1742.003, 39761.167)
7-ketolithocholic acid	1.678	0.019	9.82	1072.025 (102.056, 2290.184) ^#^	3195.725 (923.630, 46443.611)	18762.280 (3904.630, 70673.833)

*^#^ P* < 0.05 compared with HC group; *P < 0.05 compared with NCTT group.

### Correlation analysis of differential intestinal flora, fecal metabolites and clinical symptoms

**Figure 7:**
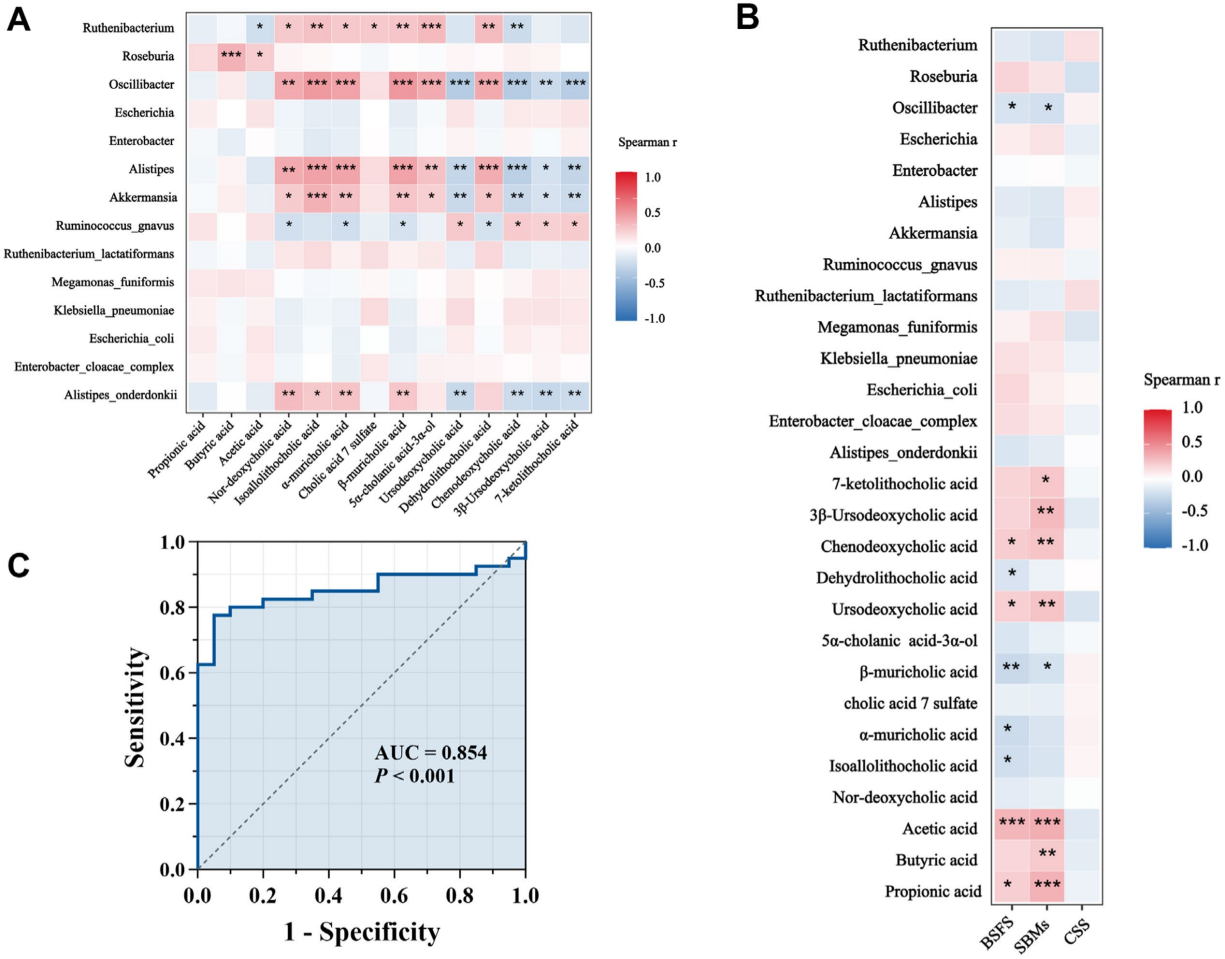
**(A)** Correlations between differential genera and differentially abundant metabolites. Red indicates a positive correlation, blue indicates a negative correlation, *p* < 0.05, ** *p* < 0.01, *** *p* < 0.001. **(B)** Correlation analysis between CSS score of clinical symptoms of constipation, BSFS score, number of spontaneous bowel movements per week, and diagnostic intestinal biomarkers; red indicates a positive correlation, blue indicates a negative correlation, *p < 0.05, **p < 0.01, ***p < 0.001. **(C)** ROC curves for different genera and metabolite combinations (*Oscillibacter*, *Alistipes*, isoallolithocholic acid, *α*-muricholic acid, *β*-muricholic acid, dehydrolithocholic acid, and nor-deoxycholic acid) with Spearman’s |r| values > 0.6.

To explore the interactions among gut microbiota, fecal metabolites, and clinical symptoms, we analyzed the correlations between 14 differential genera, 14 significantly altered metabolites, and constipation-related indicators, including the BSFS score, the number of SBMs, and the CSS score (Figures 7A,B, Supplementary Tables S3, S4). Notably, Roseburia, enriched in the NCTT group, was positively correlated with butyric acid and acetic acid, two key SCFAs known to enhance intestinal motility. In contrast, taxa enriched in the DCTT group, including Oscillibacter, Akkermansia, Alistipes, and *Alistipes onderdonkii*, showed strong negative correlations with ursodeoxycholic acid, chenodeoxycholic acid, 3*β*-ursodeoxycholic acid, and 7-ketolithocholic acid, and strong positive correlations with nor-deoxycholic acid, isoallolithocholic acid, *α*-muricholic acid, and *β*-muricholic acid—bile acids that have been associated with impaired gut motility. Clinical correlation analyses revealed that the BSFS score was positively associated with propionic acid, acetic acid, ursodeoxycholic acid, and chenodeoxycholic acid, and negatively associated with isoallolithocholic acid, α-muricholic acid, β-muricholic acid, and dehydrolithocholic acid. Similarly, the number of SBMs was positively correlated with multiple SCFAs (e.g., acetic acid, propionic acid, butyric acid) and bile acids (e.g., ursodeoxycholic acid, chenodeoxycholic acid, 3β-ursodeoxycholic acid, 7-ketolithocholic acid), and negatively correlated with β-muricholic acid. Importantly, no significant correlations were observed between the CSS score and the differential microbiota or metabolites. Collectively, these findings suggest that alterations in specific microbes and their associated metabolites, particularly SCFAs and bile acids, may contribute to differences in colonic transit and symptom among FC subtypes.

### Functional predictive analysis of FC-associated differential flora and metabolites

We plotted ROC curves for differential genera and metabolite combinations with Spearman’s |r | > 0.6 and FDR *p* < 0.05 (*Oscillibacter*, *Alistipes,* isoallolithocholic acid, *α*-muricholic acid, *β*-muricholic acid, dehydrolithocholic acid, nor-deoxycholic acid), revealing that the combination of these genera and metabolites had an AUC value of 0.854 in distinguishing FC patients from healthy individuals a general AUC value of > 0.85 is considered to be a very good predictor (Gong et al., 2021); this indicates that the interaction of these genera and metabolites is an important pathogenetic mechanism for FC intestinal dyskinesia and deserves further exploration. In addition, we calculated AUC values for 14 genera and 14 differentially abundant metabolites to further identify key genera or metabolites. The differential microbiota and differentially abundant metabolites had good predictive functions (Tables 5–8), especially metabolites such as isoallolithocholic acid, α-muricholic acid, cholic acid 7 sulfate, β-muricholic acid, and dehydrolithocholic acid. The AUC value of the flora, such as *Escherichia_coli* and *Alistipes*, was > 0.7, which could distinguish the DCTT group from the NCTT group. The metabolites nor-deoxycholic acid, isoallolithocholic acid, α-muricholic acid, cholic acid 7 sulfate, β-muricholic acid, 5α-cholanic acid-3α-ol, dehydrolithocholic acid, and flora, such as *Akkermansia*, *Alistipes*, and *Oscillibacter*, and *Ruthenibacterium*, with AUC values > 0.7, could better differentiate the DCTT group from the HC group. In particular, the AUC value of β-muricholic acid > 0.9 had the highest discriminatory ability and provided an experimental basis for the pathophysiological diagnosis of FC.

**Table 5:** AUC values of the 14 differentially abundant metabolites (DCTT vs. NCTT).

Metabolites	AUC value	95% confidence interval	*P* value
Propionic acid	0.365	0.188–0.542	0.144
Butyric acid	0.335	0.163–0.507	0.074
Acetic acid	0.28	0.115–0.445	0.017
Nor-deoxycholic acid	0.695	0.527–0.863	0.035
Isoallolithocholic acid	0.724	0.558–0.889	0.015
α-muricholic acid	0.773	0.617–0.928	0.003
Cholic acid 7 sulfate	0.728	0.565–0.89	0.014
β-muricholic acid	0.793	0.643–0.942	0.002
5α-cholanic acid-3α-ol	0.638	0.462–0.813	0.137
Ursodeoxycholic acid	0.305	0.140–0.47	0.035
Dehydrolithocholic acid	0.705	0.543–0.867	0.027
Chenodeoxycholic acid	0.190	0.053–0.327	0.001
3β-ursodeoxycholic acid	0.313	0.148–0.477	0.042
7-ketolithocholic acid	0.295	0.134–0.456	0.027

**Table 6:** AUC values of the 14 differential genera (DCTT vs. NCTT).

Microorganisms	AUC value	95% confidence interval	*P* value
*Alistipes_onderdonkii*	0.518	0.331–0.704	0.850
*Enterobacter_cloacae_complex*	0.678	0.504–0.851	0.055
*Escherichia_coli*	0.713	0.55–0.875	0.021
*Klebsiella_pneumoniae*	0.580	0.399–0.761	0.387
*Megamonas_funiformis*	0.370	0.194–0.546	0.160
*Ruthenibacterium_lactatiformans*	0.560	0.378–0.742	0.516
*Ruminococcus_gnavus*	0.445	0.262–0.628	0.552
*Akkermansia*	0.685	0.519–0.851	0.045
*Alistipes*	0.715	0.553–0.877	0.020
*Enterobacter*	0.665	0.488–0.842	0.074
*Escherichia*	0.583	0.401–0.764	0.372
*Oscillibacter*	0.658	0.485–0.830	0.088
*Roseburia*	0.210	0.054–0.366	0.002
*Ruthenibacterium*	0.690	0.524–0.856	0.040

**Table 7:** AUC values of the 14 differentially abundant metabolites (DCTT vs. HC).

Metabolites	AUC value	95% confidence interval	*P* value
Propionic acid	0.138	0.026–0.249	<0.001
Butyric acid	0.265	0.107–0.423	0.011
Acetic acid	0.050	0.000–0.120	<0.001
Nor-deoxycholic acid	0.759	0.61–0.908	0.005
Isoallolithocholic acid	0.829	0.691–0.967	<0.001
α-muricholic acid	0.835	0.700–0.970	<0.001
Cholic acid 7 sulfate	0.706	0.536–0.876	0.026
β-muricholic acid	0.903	0.792–1.000	<0.001
5α-cholanic acid-3α-ol	0.741	0.581–0.902	0.009
Ursodeoxycholic acid	0.175	0.045–0.305	<0.001
Dehydrolithocholic acid	0.765	0.616–0.914	0.004
Chenodeoxycholic acid	0.143	0.021–0.264	<0.001
3β-ursodeoxycholic acid	0.165	0.043–0.287	<0.001
7-ketolithocholic acid	0.145	0.025–0.265	<0.001

**Table 8:** AUC values of the 14 differential genera (DCTT vs. HC).

Microorganisms	AUC value	95% confidence interval	*P* value
*Alistipes_onderdonkii*	0.670	0.503–0.837	0.066
*Enterobacter_cloacae_complex*	0.373	0.194–0.551	0.168
*Escherichia_coli*	0.345	0.169–0.521	0.094
*Klebsiella_pneumoniae*	0.370	0.194–0.546	0.160
*Megamonas_funiformis*	0.381	0.200–0.563	0.199
*Ruthenibacterium_lactatiformans*	0.663	0.491–0.834	0.079
*Ruminococcus gnavus*	0.345	0.172–0.518	0.094
*Akkermansia*	0.758	0.605–0.910	0.005
*Alistipes*	0.855	0.734–0.976	<0.001
*Enterobacter*	0.353	0.179–0.526	0.110
*Escherichia*	0.313	0.146–0.479	0.042
*Oscillibacter*	0.868	0.758–0.977	<0.001
*Roseburia*	0.345	0.163–0.527	0.094
*Ruthenibacterium*	0.790	0.649–0.931	0.002

## Discussion

FC is a disease that can seriously affect an individual’s health status, greatly reduces quality of life, and is classified as intractable by the World Health Organization. Therefore, in-depth explorations of its pathogenesis are urgently needed. In recent years, the study of the correlation between the intestinal flora, metabolites, and FC has gradually become an important tool for pathological research and provides a new perspective for clinical treatment. However, the contribution of the intestinal flora and related metabolites to the pathogenesis of the function of colonic transport in FC has not been elucidated. In this study, we recruited 20 patients with FC with delayed colonic transit, 20 patients with FC with normal colonic transit time, and 20 healthy volunteers. We then examined the changes in their intestinal flora and fecal metabolites and assessed the associations between different intestinal flora and fecal metabolite interactions and different colonic transport dynamics in FC.

We found that the abundance of the intestinal flora in FC patients and healthy individuals with different colonic transport dynamics was significantly different, and the abundance of the intestinal flora in FC patients with slow colonic transport was significantly greater than that in the NCTT and HC groups. There were significant differences in the flora structure in each group at the genus and species levels, consistent with previous findings (Ge et al., 2017; Li R. et al., 2023; Li Y. Q. et al., 2023; Wang et al., 2023). These results suggest that abnormalities in intestinal dynamics affected the composition and function of the microbiota. This aligns with the “r/K selection” theory of environmental disturbances in ecology (Tipton et al., 2019), which indicates that organisms choose the appropriate method of survival for the different conditions in which they live. For example, when intestinal transport is accelerated, strains with high fecundity are better adapted to grow in a less competitive intestinal environment, called “r-selection.” In contrast, when the intestinal transport time slows, and the surrounding competitive environment increases, strains with high viability can grow slowly under unrestricted conditions, known as “K-selection.” In FC, impaired colonic motility leads to prolonged transit time, resunutrient depletion and reduced substrate availability, which subsequently favors the colonizatslow-growing, highly competitive microbial species. Moreover, the mechanistic relationship between the microbiota and colonic transport is bidirectional, with the microbiota itself regulating intestinal motility. Fecal microbiota transplantation can improve slow colonic transport and associated constipation in rodents and humans (Zhang et al., 2018).

The genera *Alistipes*, *Akkermansia*, *Oscillibacter*, *Ruthenibacterium*, *Alistipes*_*onderdonkii*, and *Ruthenibacterium_lactatiformans* were significantly enriched in patients with FC with slow colonic transit. *Roseburia* and *Klebsiella_pneumoniae* were enriched in patients with FC with normal colonic transit, *Escherichia*, *Enterobacter*, *Escherichia_coli*, *Ruminococcus gnavus*, *Enterobacter cloacae_complex*, and *Megamonas uniformis* were enriched in the HC group. *Alistipes* (Sugitani et al., 2021; Tian et al., 2022), *Akkermansia* (Li Y. Q. et al., 2023; Yi et al., 2023), *Oscillibacter* (Yao et al., 2024; Zhang X. et al., 2021), and *Ruthenibacterium lactatiformans* (Kovtun et al., 2022) are considered potential pathogens and were shown to be enriched in DCTT patients, which is consistent with the findings of previous studies. *Alistipes* is an indole-positive organism, and increased levels of this genus disrupt the cerebral–gut axis and decrease serotonin availability. Tryptophan is the precursor of 5-hydroxytryptophan, and 5-serotonin is a precursor to 5-hydroxytryptophan; a decrease in 5-hydroxytryptophan has been associated with impaired intestinal motility in STC (Parker et al., 2020; Tian et al., 2020). *Akkermansia* can excessively degrade mucins, leading to intestinal barrier dysfunction and the accumulation of harmful substances, which ultimately leads to the development of intestinal disorders (Zhang et al., 2016). *Oscillibacter*-type strains produce valeric acid, a neurotransmitter *γ*-aminobutyric acid homolog of the neurotransmitter γ-aminobutyric acid that is significantly associated with depression, as their primary metabolic end product (Cheung et al., 2019; Naseribafrouei et al., 2014). It has been shown that depression is associated with slower intestinal transport, with a prevalence of anxiety and depression of approximately 23% in patients with slow-transport constipation (Tanner et al., 2021). Furthermore, there are significant interactions between mood and gastrointestinal disorders (Haj et al., 2018). Stress can trigger gastrointestinal symptoms that exacerbate functional gastrointestinal disorders through the hypothalamic-vagal circuitry, including delayed gastric emptying (Jiang and Travagli, 2020). Additional studies have also demonstrated the occurrence of reduced proximal and distal colonic contractile peristalsis in stressed mice (Ono et al., 2022). *Ruthenibacterium lactatiformans* is a gram-negative and lactic acid-producing bacterium (Shkoporov et al., 2016) thought to contribute to disease progression in multiple sclerosis patients by triggering mitochondrial dysfunction (Esmael et al., 2021)^.^ Loperamide-induced constipation in mice has been reported to result in mitochondrial dysfunction, which can be enhanced by increasing the activity levels of oxidative stress factors and energy-producing related enzymes that can increase intestinal motility and improve constipation (Hao et al., 2023). *Roseburia*, an important genus of SCFAs (acetic acid, propionic acid, and butyric acid)-producing bacteria that breaks down non-digestible carbohydrates normally involved in energy production, can promote intestinal motility, which is significantly reduced in patients with constipation (Zhuang et al., 2019). In the present study, *Roseburia* spp. were enriched in the NCTT group and significantly reduced in the DCTT group, suggesting that reduced levels of *Roseburia* are important factors in FC-related intestinal dyskinesia and are not associated with constipation. *Klebsiella_pneumoniae*, a class of conditionally pathogenic bacteria, was enriched in the NCTT group, and alterations in the abundance of this species are either a cause or a consequence of constipation, necessitating further investigation. However, compared with those in the FC group, the relative abundances of *Megamonas_funiformi* and *Ruminococcus gnavus* were greater in the HC group, both of which belong to the phylum Thick-walled Bacteria, which can ferment a variety of carbohydrates and produce acetic acid and propionic acid (Crost et al., 2023; Tian et al., 2020), promoting intestinal peristalsis and alleviating constipation. Oral intake of *Ruminococcus gnavus* can effectively improve constipation symptoms in constipated mice transplanted with loperamide and fecal bacterium-induced constipation in constipated patients (Li R. et al., 2023). *Escherichia coli* is a gram-negative, partially anaerobic bacterium widely found in the human intestinal tract. Randomized, double-blind, placebo-controlled clinical trials have shown that *E. coli*-based probiotics are effective in relieving constipation symptoms (Mollenbrink and Bruckschen, 1994). Additionally, an increase in the relative abundance of *Escherichia* has been observed following fecal microbiota transplantation in patients with slow-transit constipation (Xie et al., 2021). *Escherichia coli* synthesizes tryptophan via tryptophan synthase, supplying an additional source of substrate for 5-Hydroxytryptamine production through enterochromaffin cells in the gut (Xiong et al., 2021). Research on *Enterobacter* in FC remains limited. In our study, the relative abundance of *Enterobacter* was lower in the constipation group than in the HC group. However, correlation analysis showed no significant associations between *Enterobacter* and SCFAs, BAs, or clinical symptoms. Therefore, the potential role of *Enterobacter* in the pathogenesis of FC warrants further investigation in future studies.

Metabolic pathways encoded by the human intestinal flora continuously communicate with the host through many bioactive metabolites, and changes in SCFAs versus BAs were observed in our study. SCFAs are produced by bacterial fermentation of dietary fibers or other components of the diet, including acetic acid, propionic acid, butyric acid, and valeric acids, and are among the most abundant microbial metabolites in the lumen of the intestines. SCFAs can regulate cell proliferation and differentiation by binding to cell surface receptors; furthermore, they can enter cells through specific transport proteins, directly affecting cellular metabolism and influencing cellular energy and signaling pathways (Zhuang et al., 2019). SCFAs stimulate intestinal contractions, thereby promoting gastrointestinal motility (Martin-Gallausiaux et al., 2021). In this study, we found that the levels of acetic acid, propionic acid, and butyric acid in the DCTT group were lower than those in the NCTT and HC groups to varying degrees. One study reported that a decrease in the levels of acetic acid, propionic acid, and butyric acid was associated with delayed transmission time in patients with constipation, and our analysis of FC patients with varying colonic transmission dynamics further demonstrated that the levels of these acids were closely related to the level of colonic transmission (Jalanka et al., 2019). BAs are considered “physiological laxatives.” Studies have demonstrated that patients with chronic constipation have significantly lower levels of both total and primary fecal BAs. Supplementing with specific BA analogs or using drugs that inhibit ileal BA reabsorption, such as elobixibat, has been shown to improve clinical symptoms in constipated patients (Nakajima et al., 2022). BAs, acting as signaling molecules, promote intestinal motility by binding to and activating both cell surface and nuclear receptors, as well as by modulating ion channel activity (Chedid et al., 2018; Keely et al., 2022). Elevated serum bile acid levels have been observed in FC rats, alongside reduced Farnesoid X receptor (FXR) expression in the liver and ileum, enhanced bile acid reabsorption, and reduced bile acid concentrations in the colon, leading to impaired gut motility (Yin et al., 2024). In this study, we found that the primary BA levels in the DCTT group were lower compared to the NCTT and HC groups, with no significant differences in total and secondary BAs across the three groups, although a decreasing trend was noted in the DCTT group. Additionally, we observed increased levels of *β*-muricholic acid and *α*-muricholic acid in the DCTT group, whereas chenodeoxycholic acid levels were reduced, and these findings were correlated with clinical symptoms. FXR interacts with various endogenous BAs, with chenodeoxycholic acid being the most potent FXR activator, while muricholic acid acts as an FXR antagonist (Gonzalez et al., 2016). These results suggest that FXR function in the intestines of constipated patients may be impaired, contributing to BA metabolic disturbances that affect gut motility. Further investigations are required to explore these mechanisms in greater detail.

Harder stools and fewer bowel movements are usually caused by reduced colonic transit times, which affects the majority of FC patients with slow colonic transit. We used the BSFS score with the number of SBMs as a noninvasive proxy for colonic transit time and analyzed the correlation of the 14 differential bacterial genera and metabolites in this cohort with the clinical symptoms and severity of FC. *Oscillibacter*, isoallolithocholic acid, α-muricholic acid, β-muricholic acid, and dehydrolithocholic acid was significantly associated with harder feces and fewer bowel movements. Lower levels of acetic acid, propionic acid, ursodeoxycholic acid, and chenodeoxycholic acid were significantly associated with harder feces and fewer bowel movements and positively correlated with constipation severity. Butyric acid was significantly associated with fewer bowel movements. The combination of differential bacteria and metabolites (*Oscillibacter*, *Alistipes*, isoallolithocholic acid, α-muricholic acid, β-muricholic acid, dehydrolithocholic acid, and nor-deoxycholic acid) effectively distinguished patients with FC from healthy individuals (AUC:0.854) and played a critical role in diagnostic assessment. In particular, *Oscillibacter* and the metabolite β-muricholic acid show the best diagnostic efficacy for DCTT and are expected to become clinical diagnostic biomarkers for DCTT. Moreover, it is noteworthy that in this study, patients with delayed colonic transit, exhibited a reduction in *Ruminococcus gnavus* and *Roseburia*, two well-known butyrate-producing genera. Correspondingly, the levels of SCFAs, such as acetate, propionate, and butyrate, as well as BAs such as chenodeoxycholic acid, were significantly decreased. The identified microbial and metabolic signatures provide promising opportunities for clinical interventions, and may serve as potential targets for personalized therapeutic strategies, such as butyrate supplementation, BA modulation, or customized probiotic therapies aimed at restoring beneficial genera like *Ruminococcus gnavus* and *Roseburia*. Future studies are warranted to explore the causal relationships between these alterations and colonic transit dysfunction, and to evaluate the efficacy of microbiota- or metabolite-based therapies in improving gut motility disorders and clinical outcomes in FC patients.

KEGG functional analysis revealed that several key metabolic pathways related to energy production and neurotransmission—including fructose and mannose metabolism, galactose metabolism, pentose and glucuronate interconversions, pyruvate metabolism, propanoate metabolism, and serotonergic synapse—were significantly downregulated in the DCTT group compared to the NCTT and HC groups. Fructose levels were positively correlated with propionate, suggesting its role in promoting propionate metabolism, while galactose was more strongly associated with butyrate production (Zhang et al., 2025). Pyruvate serves as a central intermediate linking glycolysis to the microbial synthesis of acetate, propionate, and butyrate via distinct fermentation pathways, and thus plays a pivotal role in maintaining gut homeostasis (Koh et al., 2016). SCFAs, particularly butyrate and propionate, can directly stimulate enterochromaffin cells in the gut epithelium by enhancing the expression of tryptophan hydroxylase 1, thereby promoting serotonin biosynthesis and accelerating intestinal peristalsis (Agus et al., 2018; Vincent et al., 2018). Moreover, 5-HT not only modulates local gut motility but also participates in gut–brain axis signaling via vagal and other neurohumoral pathways (Layunta et al., 2021). Additionally, multiple pathways associated with amino acid biosynthesis and fermentation—such as valine, leucine, and isoleucine biosynthesis, phenylalanine, tyrosine, and tryptophan biosynthesis, arginine and proline metabolism, and phenylalanine metabolism—were also downregulated in the DCTT group, consistent with previous findings (Liu et al., 2022; Wang et al., 2023; Yang et al., 2022). Most dietary proteins and peptides are normally digested in the small intestine into free amino acids, which are then absorbed into the circulation. However, undigested proteins that escape digestion in the upper gut reach the colon, where they serve as fermentable substrates for the gut microbiota and are hydrolyzed into amino acids. These colonic amino acids can subsequently be metabolized by microbes into ammonia, SCFAs, or branched-chain fatty acids (Liu et al., 2020). Collectively, these findings suggest that impaired microbial fermentation activity and potential dysfunction in gut-brain communication pathways may underlie delayed colonic transit in patients with FC. Nevertheless, it should be noted that KEGG-based microbial functional annotation represents a predictive analysis; more precise functional information requires further experimental validation.

This study has several limitations. (1) The sample size was small, and the participants all lived in the Fujian area for a long period; thus, selection bias may be present in the results. (2) The composition of the intestinal flora is closely related to age; it tends to stabilize in adulthood and gradually decrease in old age. However, this study lacked an in-depth exploration of the differences in the intestinal flora related to aging due to the insufficient sample size. (3) There are differences in the composition of the microbiota across different parts of the human gut (Chinda et al., 2022). The lateral distribution of the intestinal flora includes the lumen, mucus layer, and epithelial layer (Sartor, 2015). The lumen and mucosa-associated microbiota were distinct ecosystems with different microbial compositions as well as metabolic and immune functions (Nowicki et al., 2023), and another study revealed that FC patients had a unique colon mucosal microbiota profile, distinguishing between constipated patients and healthy individuals (Parthasarathy et al., 2016; Sugitani et al., 2021). Future studies should involve larger, multicenter cohorts across different ages and diets, focus on both luminal- and mucosa-associated microbiota, and prioritize absolute quantification to improve the accuracy and interpretation of changes in microbiota.

## Conclusion

The present analysis revealed differences in the intestinal flora and metabolites of FC patients with different colonic transmission dynamics. The gut flora richness index was elevated in FC patients with slow colonic transmission, and further screening for differential gut flora and metabolite types that may have diagnostic value may provide clues to better understand the mechanisms of intestinal microenvironmental changes in FC colonic conduction dysfunction. However, further experiments are still needed to confirm their crosstalk. Metabolite abnormalities caused by intestinal flora disorders appear to be important in the pathogenesis of FC-related intestinal dysfunction and deserve further exploration. This study provided insights into the relationships among the fecal microbiota, metabolites, and intestinal dysfunction in FC patients with different colonic transport dynamics and offers possible ideas for the diagnosis of FC and interventions targeting specific microbiota associated with the metabolism of SCFAs and BAs. These efforts may contribute to the discovery of new therapeutic interventions for individuals affected by FC.

## Data Availability

The original contributions presented in the study are publicly available. This data can be found at: https://www.ncbi.nlm.nih.gov/, accession number: PRJNA1232352.

## Glossary

AUC: area under the curve
BAs: bile acids
BSFS: Bristol Stool Form Scale
CSS: Constipation Severity Score
DCTT: delayed colonic transit time
FC: functional constipation
FDR: false discovery rate
FXR: Farnesoid X receptor
HC: healthy control
IBS: irritable bowel syndrome
KEGG: Kyoto Encyclopedia of Genes and Genomes
LDA: linear discriminant analysis
LSD: least significant difference
MTBE: methyl tert-butyl ether
NCTT: normal colonic transit time
OPLS–DA: orthogonal partial least squares discriminant analysis
ORF: open reading frame
PCoA: principal coordinate analysis
QTRAP: quadrupole-linear ion trap
SBMs: spontaneous bowel movements
SCFAs: short-chain fatty acids
VIP: variable importance in projection
