# Supplementary material for: Correlation of the intestinal flora and its metabolites with the colonic transport function in functional constipation
Source: Front Microbiol. 2025 May 21;16:1591697. doi: 10.3389/fmicb.2025.1591697 (PMC12134075; doi:10.3389/fmicb.2025.1591697)
Supplement: Supplementary file 1 [file Supplementary_file_1.docx]

Supplementary Material

# Supplementary Figures and Tables

## Supplementary Tables S1.Table of data quality assessment of sequencing outputs for each sample

| **Sample** | **RawReads** | **RawBases** | **CleanReads** | **CleanBases**  **(bp)** | **CleanQ20(%)** | **CleanQ30(%)** | **CleanGC(%)** |
| --- | --- | --- | --- | --- | --- | --- | --- |
| DCTT1 | 95213360 | 14282004000 | 70430952 | 10458712422 | 100 | 99.72 | 48.00 |
| DCTT2 | 98304868 | 14745730200 | 70450286 | 10462331863 | 100 | 99.73 | 50.50 |
| DCTT3 | 85890932 | 12883639800 | 63351430 | 9408609413 | 100 | 99.75 | 52.00 |
| DCTT4 | 81257260 | 12188589000 | 61395962 | 9109991442 | 100 | 99.72 | 46.00 |
| DCTT5 | 66518366 | 9977754900 | 48146796 | 7137070135 | 100 | 99.66 | 47.00 |
| DCTT6 | 71424750 | 10713712500 | 51478502 | 7638926203 | 100 | 99.69 | 51.00 |
| DCTT7 | 80750128 | 12112519200 | 57488752 | 8540009596 | 100 | 99.71 | 48.00 |
| DCTT8 | 67257516 | 10088627400 | 48037648 | 7134906725 | 100 | 99.72 | 47.00 |
| DCTT9 | 97437888 | 14615683200 | 73700646 | 10949793610 | 100 | 99.72 | 47.00 |
| DCTT10 | 103461862 | 15519279300 | 76075444 | 11301498827 | 100 | 99.70 | 47.00 |
| DCTT11 | 115736580 | 17360487000 | 85284410 | 12675344621 | 100 | 99.74 | 48.00 |
| DCTT12 | 69355468 | 10403320200 | 49929038 | 7421161065 | 100 | 99.80 | 50.00 |
| DCTT13 | 95028618 | 14254292700 | 70459668 | 10474894827 | 100 | 99.79 | 47.00 |
| DCTT14 | 94410546 | 14161581900 | 68153682 | 10127360165 | 100 | 99.80 | 47.00 |
| DCTT15 | 123547422 | 18532113300 | 90104710 | 13391118787 | 100 | 99.80 | 50.50 |
| DCTT16 | 96378944 | 14456841600 | 74977154 | 11150290214 | 100 | 99.75 | 50.00 |
| DCTT17 | 69561174 | 10434176100 | 53016972 | 7886496959 | 100 | 99.75 | 49.00 |
| DCTT18 | 129454402 | 19418160300 | 98513656 | 14656125173 | 100 | 99.75 | 48.00 |
| DCTT19 | 89005730 | 13350859500 | 70558262 | 10492945563 | 100 | 99.74 | 44.00 |
| DCTT20 | 81127484 | 12169122600 | 60891238 | 9052195108 | 100 | 99.72 | 47.00 |
| NCTT1 | 89656652 | 13448497800 | 66934072 | 9938990958 | 100 | 99.73 | 47.00 |
| NCTT2 | 76801932 | 11520289800 | 57567626 | 8554678652 | 100 | 99.74 | 46.50 |
| NCTT3 | 93793724 | 14069058600 | 70836506 | 10516374307 | 100 | 99.73 | 48.00 |
| NCTT4 | 99494068 | 14924110200 | 74596220 | 11086919622 | 100 | 99.74 | 45.00 |
| NCTT5 | 92546348 | 13881952200 | 69777238 | 10373124513 | 100 | 99.75 | 49.00 |
| NCTT6 | 116603416 | 17490512400 | 86139744 | 12810772216 | 100 | 99.81 | 46.00 |
| NCTT7 | 121320066 | 18198009900 | 87202132 | 12953653391 | 100 | 99.78 | 51.00 |
| NCTT8 | 117482016 | 17622302400 | 87383476 | 12984928198 | 100 | 99.79 | 47.00 |
| NCTT9 | 108286036 | 16242905400 | 75353730 | 11195110678 | 100 | 99.78 | 45.00 |
| NCTT10 | 118854118 | 17828117700 | 92129910 | 13704485926 | 100 | 99.76 | 52.00 |
| NCTT11 | 81275482 | 12191322300 | 63283340 | 9416294449 | 100 | 99.75 | 46.00 |
| NCTT12 | 66704492 | 10005673800 | 49984804 | 7432624955 | 100 | 99.72 | 56.00 |
| NCTT13 | 99257102 | 14888565300 | 77278666 | 11501675879 | 100 | 99.77 | 47.00 |
| NCTT14 | 96927362 | 14539104300 | 74654692 | 11108729560 | 100 | 99.75 | 47.50 |
| NCTT15 | 68318730 | 10247809500 | 52826456 | 7863240868 | 100 | 99.77 | 46.00 |
| NCTT16 | 95877632 | 14381644800 | 74560990 | 11086874447 | 100 | 99.73 | 51.00 |
| NCTT17 | 108822622 | 16323393300 | 84455236 | 12571046731 | 100 | 99.77 | 47.00 |
| NCTT18 | 88612204 | 13291830600 | 68318060 | 10165992598 | 100 | 99.76 | 47.00 |
| NCTT19 | 112697248 | 16904587200 | 87561928 | 13016690167 | 100 | 99.73 | 50.00 |
| NCTT20 | 96539880 | 14480982000 | 76018172 | 11310701646 | 100 | 99.75 | 46.00. |
| HC1 | 74731426 | 11209713900 | 57725918 | 8591649135 | 100 | 99.76 | 49.00 |
| HC2 | 114443128 | 17166469200 | 87271028 | 12978842787 | 100 | 99.75 | 50.00 |
| HC3 | 114589040 | 17188356000 | 89566114 | 13323064360 | 100 | 99.77 | 46.00 |
| HC4 | 84760400 | 12714060000 | 66659364 | 9910460928 | 100 | 99.72 | 45.00 |
| HC5 | 109911356 | 16486703400 | 85983470 | 12787771637 | 100 | 99.75 | 46.50 |
| HC6 | 94430088 | 14164513200 | 73619450 | 10951237291 | 100 | 99.76 | 47.00 |
| HC7 | 112356930 | 16853539500 | 86577432 | 12864660363 | 100 | 99.71 | 49.00 |
| HC8 | 123191922 | 18478788300 | 95731590 | 14240190943 | 100 | 99.75 | 47.50 |
| HC9 | 82715610 | 12407341500 | 61104792 | 9080188892 | 100 | 99.72 | 52.50 |
| HC10 | 81893424 | 12284013600 | 63737938 | 9482588215 | 100 | 99.75 | 47.00 |
| HC11 | 67125230 | 10068784500 | 51134058 | 7598544605 | 100 | 99.72 | 43.00 |
| HC12 | 88090520 | 13213578000 | 69148060 | 10293404651 | 100 | 99.78 | 46.00 |
| HC13 | 83474666 | 12521199900 | 65952918 | 9806946119 | 100 | 99.74 | 47.00 |
| HC14 | 94891058 | 14233658700 | 73861846 | 10989274079 | 100 | 99.75 | 45.00 |
| HC15 | 76692804 | 11503920600 | 60056136 | 8935241280 | 100 | 99.75 | 46.00 |
| HC16 | 120993970 | 18149095500 | 94020100 | 13991457019 | 100 | 99.78 | 50.00 |
| HC17 | 116681956 | 17502293400 | 92085668 | 13705970136 | 100 | 99.78 | 47.00 |
| HC18 | 118789364 | 17818404600 | 92071804 | 13706454214 | 100 | 99.79 | 45.00 |
| HC19 | 86671718 | 13000757700 | 68630714 | 10196301039 | 100 | 99.75 | 44.00 |
| HC20 | 90717862 | 13607679300 | 70292966 | 10443856232 | 100 | 99.73 | 45.00 |
| Note:Raw Reads:Number of sequencing reads generated from raw image data via base calling.Raw Bases:Total number of bases contained in the raw sequencing data.Clean Reads:Number of high-quality reads retained after filtering low-quality and contaminant sequences from the raw reads.Clean Bases:Total number of bases remaining after quality filtering.Clean Q20 (%):Percentage of bases in the clean data with a Phred score ≥20 (error rate <1%).  Clean Q30 (%):Percentage of bases in the clean data with a Phred score ≥30 (error rate <0.1%).Clean GC Content (%):Proportion of guanine (G) and cytosine (C) bases in the clean data as a percentage of total bases.control, indicating sequencing efficiency. | | | | | | | |

## Supplementary Tables S2.Table of data quality assessment of sequencing outputs for each sample

| **Sample** | **Total number** | **Total len(bp)** | **Average len(bp)** | **Max len(bp)** | **N50 len.(bp)** | **N90 len(bp)** | **GC(%)** |
| --- | --- | --- | --- | --- | --- | --- | --- |
| DCTT1 | 67574 | 155961719 | 2308.01 | 572861 | 7821 | 728 | 49.34 |
| DCTT2 | 160479 | 336405757 | 2096.26 | 349531 | 4596 | 710 | 49.69 |
| DCTT3 | 85219 | 187141198 | 2196.00 | 587287 | 5763 | 711 | 48.65 |
| DCTT4 | 58873 | 189014450 | 3210.55 | 503930 | 17563 | 887 | 46.11 |
| DCTT5 | 97509 | 190415296 | 1952.80 | 363865 | 4224 | 665 | 49.71 |
| DCTT6 | 102805 | 223784855 | 2176.79 | 520856 | 5210 | 709 | 48.26 |
| DCTT7 | 136918 | 277410238 | 2026.10 | 326127 | 4311 | 690 | 48.78 |
| DCTT8 | 86283 | 221226866 | 2563.97 | 310202 | 8531 | 768 | 47.63 |
| DCTT9 | 93805 | 237660280 | 2533.56 | 402732 | 8396 | 775 | 48.74 |
| DCTT10 | 186392 | 374517267 | 2009.30 | 467729 | 3979 | 693 | 47.90 |
| DCTT11 | 174808 | 396330849 | 2267.24 | 555061 | 6569 | 723 | 47.55 |
| DCTT12 | 117573 | 267307589 | 2273.55 | 368421 | 5232 | 749 | 48.79 |
| DCTT13 | 91645 | 213396429 | 2328.51 | 593099 | 6534 | 745 | 49.17 |
| DCTT14 | 124710 | 282778474 | 2267.49 | 432626 | 5921 | 730 | 48.36 |
| DCTT15 | 131688 | 322813002 | 2451.35 | 475952 | 7022 | 766 | 49.61 |
| DCTT16 | 153214 | 325055196 | 2121.58 | 719122 | 4770 | 716 | 47.65 |
| DCTT17 | 86385 | 232499947 | 2691.44 | 617862 | 9267 | 811 | 47.12 |
| DCTT18 | 96550 | 239254000 | 2478.03 | 536333 | 7463 | 779 | 47.79 |
| DCTT19 | 46034 | 121213756 | 2633.14 | 548210 | 9093 | 795 | 47.09 |
| DCTT20 | 163366 | 349443510 | 2139.02 | 633618 | 4661 | 717 | 49.04 |
| NCTT1 | 141401 | 318438538 | 2252.02 | 417494 | 5955 | 726 | 47.67 |
| NCTT2 | 101755 | 238091904 | 2339.85 | 280141 | 5373 | 779 | 46.67 |
| NCTT3 | 128848 | 280902865 | 2180.11 | 630685 | 5758 | 706 | 48.34 |
| NCTT4 | 90204 | 226256328 | 2508.27 | 685471 | 7802 | 778 | 46.82 |
| NCTT5 | 92247 | 235532322 | 2553.28 | 459178 | 7701 | 790 | 48.12 |
| NCTT6 | 89286 | 257165250 | 2880.24 | 360940 | 11807 | 857 | 47.40 |
| NCTT7 | 172524 | 394483501 | 2286.54 | 435016 | 6341 | 734 | 49.26 |
| NCTT8 | 105767 | 287875787 | 2721.79 | 623792 | 9967 | 806 | 45.49 |
| NCTT9 | 36481 | 165064931 | 4524.68 | 419395 | 21446 | 1385 | 47.70 |
| NCTT10 | 165808 | 360011996 | 2171.26 | 480301 | 4978 | 725 | 50.50 |
| NCTT11 | 85464 | 231416697 | 2707.77 | 481758 | 8231 | 830 | 46.30 |
| NCTT12 | 22046 | 49428750 | 2242.07 | 671462 | 5755 | 728 | 45.14 |
| NCTT13 | 73018 | 208026395 | 2848.97 | 586105 | 10397 | 866 | 46.34 |
| NCTT14 | 152159 | 370836238 | 2437.16 | 397270 | 6701 | 770 | 47.48 |
| NCTT15 | 31582 | 123624759 | 3914.41 | 608643 | 27871 | 1083 | 46.79 |
| NCTT16 | 61992 | 166526151 | 2686.25 | 679667 | 10349 | 801 | 46.09 |
| NCTT17 | 93630 | 234661180 | 2506.26 | 264885 | 8152 | 773 | 47.09 |
| NCTT18 | 91320 | 247252986 | 2707.54 | 359487 | 8403 | 824 | 46.13 |
| NCTT19 | 194930 | 445797556 | 2286.96 | 344532 | 6021 | 727 | 48.95 |
| NCTT20 | 103246 | 242424233 | 2348.03 | 428198 | 7136 | 745 | 46.72 |
| HC1 | 132416 | 263460352 | 1989.64 | 344162 | 3939 | 690 | 48.37 |
| HC2 | 119278 | 264491645 | 2217.44 | 646654 | 5528 | 729 | 47.62 |
| HC3 | 108756 | 260868245 | 2398.66 | 466980 | 8003 | 741 | 46.09 |
| HC4 | 66234 | 192304652 | 2903.41 | 320605 | 10872 | 872 | 46.93 |
| HC5 | 78731 | 210492604 | 2673.57 | 354068 | 8204 | 834 | 48.15 |
| HC6 | 140833 | 329721477 | 2341.22 | 841513 | 5452 | 773 | 46.81 |
| HC7 | 151878 | 340333094 | 2240.83 | 290722 | 5197 | 743 | 47.94 |
| HC8 | 152547 | 367012935 | 2405.90 | 516996 | 6556 | 768 | 46.94 |
| HC9 | 45925 | 114334187 | 2489.58 | 375619 | 7547 | 775 | 46.01 |
| HC10 | 117560 | 260042339 | 2212.00 | 463972 | 4796 | 745 | 46.81 |
| HC11 | 57158 | 166057708 | 2905.24 | 330882 | 10524 | 861 | 45.95 |
| HC12 | 49951 | 168428557 | 3371.88 | 366428 | 15054 | 988 | 46.80 |
| HC13 | 146300 | 300028741 | 2050.78 | 311345 | 4096 | 712 | 47.70 |
| HC14 | 60510 | 170189303 | 2812.58 | 535721 | 11096 | 827 | 45.70 |
| HC15 | 60506 | 153747109 | 2541.02 | 317042 | 7898 | 782 | 46.69 |
| HC16 | 89975 | 206655244 | 2296.81 | 337368 | 5434 | 753 | 47.53 |
| HC17 | 43459 | 120388821 | 2770.17 | 985712 | 13403 | 815 | 44.58 |
| HC18 | 72289 | 185560727 | 2566.93 | 675945 | 11343 | 745 | 46.59 |
| HC19 | 53930 | 167906076 | 3113.41 | 405786 | 15477 | 871 | 45.16 |
| HC20 | 74760 | 178116845 | 2382.52 | 681411 | 6773 | 768 | 46.89 |
| Note:Total Number: The total number of scaftigs generated from individual de novo assemblies of each sample.Total Length: The cumulative length of all scaftigs obtained from the individual assemblies of each sample.Average Length: The average length of scaftigs for each sample, calculated as the total length divided by the number of scaftigs.Max Length: The length of the longest scaftig obtained from the assembly of each sample.N50: The scaftig length at which 50% of the total assembled nucleotide length is contained in scaftigs of that length or longer. caftigs are sorted from longest to shortest, and lengths are cumulatively summed until the halfway point is reached; the length of the last scaftig included in this sum is the N50.N90: Similar to N50, but representing the scaftig length at which 90% of the total nucleotide length is covered.GC Content (%): The percentage of guanine (G) and cytosine (C) bases among the total base composition of all scaftigs. | | | | | | | |

**Supplementary Table S3.** Correlation analysis between 14 differential metabolites and 14 differential microbiota.

| microbiota | Metabolites | r-value | *P*-value | FDR-value |
| --- | --- | --- | --- | --- |
| *Alistipes_onderdonkii* | Propionic acid | -0.224 | 8.48E-02 | 2.31E-01 |
| *Alistipes_onderdonkii* | Butyric acid | -0.006 | 9.66E-01 | 9.94E-01 |
| *Alistipes_onderdonkii* | Acetic acid | -0.209 | 1.10E-01 | 2.91E-01 |
| *Alistipes_onderdonkii* | Nor-deoxycholic acid | 0.479 | 1.07E-04 | 1.10E-03 |
| *Alistipes_onderdonkii* | Isoallolithocholic acid | 0.383 | 2.51E-03 | 1.18E-02 |
| *Alistipes_onderdonkii* | α-muricholic acid | 0.420 | 8.31E-04 | 5.82E-03 |
| *Alistipes_onderdonkii* | Cholic acid 7 sulfate | -0.093 | 4.78E-01 | 6.48E-01 |
| *Alistipes_onderdonkii* | β-muricholic acid | 0.397 | 1.68E-03 | 8.67E-03 |
| *Alistipes_onderdonkii* | 5α-cholanic acid-3α-ol | 0.163 | 2.15E-01 | 4.01E-01 |
| *Alistipes_onderdonkii* | Ursodeoxycholic acid | -0.430 | 6.04E-04 | 4.38E-03 |
| *Alistipes_onderdonkii* | Dehydrolithocholic acid | 0.306 | 1.72E-02 | 5.83E-02 |
| *Alistipes_onderdonkii* | Chenodeoxycholic acid | -0.398 | 1.62E-03 | 8.60E-03 |
| *Alistipes_onderdonkii* | 3β-ursodeoxycholic acid | -0.444 | 3.83E-04 | 3.27E-03 |
| *Alistipes_onderdonkii* | 7-ketolithocholic acid | -0.405 | 1.35E-03 | 7.55E-03 |
| *Enterobacter_cloacae_complex* | Propionic acid | 0.089 | 5.01E-01 | 6.72E-01 |
| *Enterobacter_cloacae_complex* | Butyric acid | -0.084 | 5.25E-01 | 6.81E-01 |
| *Enterobacter_cloacae_complex* | Acetic acid | 0.134 | 3.06E-01 | 4.84E-01 |
| *Enterobacter_cloacae_complex* | Nor-deoxycholic acid | -0.104 | 4.29E-01 | 6.27E-01 |
| *Enterobacter_cloacae_complex* | Isoallolithocholic acid | -0.002 | 9.88E-01 | 1.00E+00 |
| *Enterobacter_cloacae_complex* | α-muricholic acid | -0.169 | 1.96E-01 | 3.84E-01 |
| *Enterobacter_cloacae_complex* | Cholic acid 7 sulfate | 0.164 | 2.10E-01 | 3.95E-01 |
| *Enterobacter_cloacae_complex* | β-muricholic acid | -0.145 | 2.69E-01 | 4.47E-01 |
| *Enterobacter_cloacae_complex* | 5α-cholanic acid-3α-ol | 0.095 | 4.71E-01 | 6.48E-01 |
| *Enterobacter_cloacae_complex* | Ursodeoxycholic acid | 0.087 | 5.10E-01 | 6.80E-01 |
| *Enterobacter_cloacae_complex* | Dehydrolithocholic acid | 0.073 | 5.80E-01 | 7.10E-01 |
| *Enterobacter_cloacae_complex* | Chenodeoxycholic acid | 0.023 | 8.64E-01 | 9.25E-01 |
| *Enterobacter_cloacae_complex* | 3β-ursodeoxycholic acid | 0.072 | 5.84E-01 | 7.11E-01 |
| *Enterobacter_cloacae_complex* | 7-ketolithocholic acid | 0.024 | 8.54E-01 | 9.20E-01 |
| *Escherichia_coli* | Propionic acid | 0.143 | 2.76E-01 | 4.51E-01 |
| *Escherichia_coli* | Butyric acid | -0.062 | 6.39E-01 | 7.41E-01 |
| *Escherichia_coli* | Acetic acid | 0.171 | 1.92E-01 | 3.84E-01 |
| *Escherichia_coli* | Nor-deoxycholic acid | -0.160 | 2.23E-01 | 4.06E-01 |
| *Escherichia_coli* | Isoallolithocholic acid | -0.054 | 6.83E-01 | 7.78E-01 |
| *Escherichia_coli* | α-muricholic acid | -0.168 | 2.00E-01 | 3.85E-01 |
| *Escherichia_coli* | Cholic acid 7 sulfate | -0.007 | 9.56E-01 | 9.92E-01 |
| *Escherichia_coli* | β-muricholic acid | -0.192 | 1.41E-01 | 3.31E-01 |
| *Escherichia_coli* | 5α-cholanic acid-3α-ol | -0.097 | 4.61E-01 | 6.41E-01 |
| *Escherichia_coli* | Ursodeoxycholic acid | 0.132 | 3.15E-01 | 4.92E-01 |
| *Escherichia_coli* | Dehydrolithocholic acid | -0.053 | 6.89E-01 | 7.81E-01 |
| *Escherichia_coli* | Chenodeoxycholic acid | 0.132 | 3.16E-01 | 4.92E-01 |
| *Escherichia_coli* | 3β-ursodeoxycholic acid | 0.081 | 5.39E-01 | 6.90E-01 |
| *Escherichia_coli* | 7-ketolithocholic acid | 0.136 | 3.00E-01 | 4.78E-01 |
| *Klebsiella_pneumoniae* | Propionic acid | 0.098 | 4.56E-01 | 6.39E-01 |
| *Klebsiella_pneumoniae* | Butyric acid | -0.075 | 5.69E-01 | 7.06E-01 |
| *Klebsiella_pneumoniae* | Acetic acid | 0.093 | 4.77E-01 | 6.48E-01 |
| *Klebsiella_pneumoniae* | Nor-deoxycholic acid | -0.175 | 1.81E-01 | 3.73E-01 |
| *Klebsiella_pneumoniae* | Isoallolithocholic acid | -0.100 | 4.47E-01 | 6.35E-01 |
| *Klebsiella_pneumoniae* | α-muricholic acid | -0.147 | 2.64E-01 | 4.44E-01 |
| *Klebsiella_pneumoniae* | Cholic acid 7 sulfate | 0.255 | 4.95E-02 | 1.49E-01 |
| *Klebsiella_pneumoniae* | β-muricholic acid | -0.169 | 1.98E-01 | 3.84E-01 |
| *Klebsiella_pneumoniae* | 5α-cholanic acid-3α-ol | 0.044 | 7.40E-01 | 8.34E-01 |
| *Klebsiella_pneumoniae* | Ursodeoxycholic acid | 0.245 | 5.94E-02 | 1.71E-01 |
| *Klebsiella_pneumoniae* | Dehydrolithocholic acid | -0.025 | 8.50E-01 | 9.20E-01 |
| *Klebsiella_pneumoniae* | Chenodeoxycholic acid | 0.178 | 1.74E-01 | 3.67E-01 |
| *Klebsiella_pneumoniae* | 3β-ursodeoxycholic acid | 0.194 | 1.38E-01 | 3.31E-01 |
| *Klebsiella_pneumoniae* | 7-ketolithocholic acid | 0.159 | 2.26E-01 | 4.06E-01 |
| *Megamonas_funiformis* | Propionic acid | 0.155 | 2.37E-01 | 4.15E-01 |
| *Megamonas_funiformis* | Butyric acid | 0.186 | 1.54E-01 | 3.46E-01 |
| *Megamonas_funiformis* | Acetic acid | 0.156 | 2.35E-01 | 4.15E-01 |
| *Megamonas_funiformis* | Nor-deoxycholic acid | -0.032 | 8.10E-01 | 8.92E-01 |
| *Megamonas_funiformis* | Isoallolithocholic acid | -0.077 | 5.60E-01 | 6.99E-01 |
| *Megamonas_funiformis* | α-muricholic acid | -0.068 | 6.04E-01 | 7.26E-01 |
| *Megamonas_funiformis* | Cholic acid 7 sulfate | 0.056 | 6.71E-01 | 7.69E-01 |
| *Megamonas_funiformis* | β-muricholic acid | -0.085 | 5.16E-01 | 6.81E-01 |
| *Megamonas_funiformis* | 5α-cholanic acid-3α-ol | 0.013 | 9.20E-01 | 9.72E-01 |
| *Megamonas_funiformis* | Ursodeoxycholic acid | 0.123 | 3.49E-01 | 5.34E-01 |
| *Megamonas_funiformis* | Dehydrolithocholic acid | 0.012 | 9.29E-01 | 9.73E-01 |
| *Megamonas_funiformis* | Chenodeoxycholic acid | 0.069 | 6.00E-01 | 7.25E-01 |
| *Megamonas_funiformis* | 3β-ursodeoxycholic acid | 0.173 | 1.87E-01 | 3.82E-01 |
| *Megamonas_funiformis* | 7-ketolithocholic acid | 0.138 | 2.91E-01 | 4.68E-01 |
| *Ruthenibacterium_lactatiformans* | Propionic acid | -0.093 | 4.80E-01 | 6.48E-01 |
| *Ruthenibacterium_lactatiformans* | Butyric acid | -0.028 | 8.29E-01 | 9.06E-01 |
| *Ruthenibacterium_lactatiformans* | Acetic acid | -0.166 | 2.04E-01 | 3.88E-01 |
| *Ruthenibacterium_lactatiformans* | Nor-deoxycholic acid | 0.170 | 1.95E-01 | 3.84E-01 |
| *Ruthenibacterium_lactatiformans* | Isoallolithocholic acid | 0.253 | 5.11E-02 | 1.52E-01 |
| *Ruthenibacterium_lactatiformans* | α-muricholic acid | 0.102 | 4.39E-01 | 6.29E-01 |
| *Ruthenibacterium_lactatiformans* | Cholic acid 7 sulfate | 0.234 | 7.19E-02 | 1.98E-01 |
| *Ruthenibacterium_lactatiformans* | β-muricholic acid | 0.104 | 4.27E-01 | 6.27E-01 |
| *Ruthenibacterium_lactatiformans* | 5α-cholanic acid-3α-ol | 0.154 | 2.39E-01 | 4.15E-01 |
| *Ruthenibacterium_lactatiformans* | Ursodeoxycholic acid | -0.181 | 1.65E-01 | 3.60E-01 |
| *Ruthenibacterium_lactatiformans* | Dehydrolithocholic acid | 0.279 | 3.12E-02 | 1.00E-01 |
| *Ruthenibacterium_lactatiformans* | Chenodeoxycholic acid | -0.203 | 1.19E-01 | 3.11E-01 |
| *Ruthenibacterium_lactatiformans* | 3β-ursodeoxycholic acid | -0.156 | 2.33E-01 | 4.15E-01 |
| *Ruthenibacterium_lactatiformans* | 7-ketolithocholic acid | -0.179 | 1.71E-01 | 3.63E-01 |
| *Ruminococcus_gnavus* | Propionic acid | 0.192 | 1.42E-01 | 3.31E-01 |
| *Ruminococcus_gnavus* | Butyric acid | -0.005 | 9.68E-01 | 9.94E-01 |
| *Ruminococcus_gnavus* | Acetic acid | 0.196 | 1.34E-01 | 3.31E-01 |
| *Ruminococcus_gnavus* | Nor-deoxycholic acid | -0.354 | 5.59E-03 | 2.19E-02 |
| *Ruminococcus_gnavus* | Isoallolithocholic acid | -0.306 | 1.76E-02 | 5.83E-02 |
| *Ruminococcus_gnavus* | α-muricholic acid | -0.369 | 3.72E-03 | 1.55E-02 |
| *Ruminococcus_gnavus* | Cholic acid 7 sulfate | -0.169 | 1.96E-01 | 3.84E-01 |
| *Ruminococcus_gnavus* | β-muricholic acid | -0.317 | 1.35E-02 | 4.67E-02 |
| *Ruminococcus_gnavus* | 5α-cholanic acid-3α-ol | -0.154 | 2.41E-01 | 4.15E-01 |
| *Ruminococcus_gnavus* | Ursodeoxycholic acid | 0.383 | 2.53E-03 | 1.18E-02 |
| *Ruminococcus_gnavus* | Dehydrolithocholic acid | -0.330 | 9.97E-03 | 3.62E-02 |
| *Ruminococcus_gnavus* | Chenodeoxycholic acid | 0.370 | 3.65E-03 | 1.55E-02 |
| *Ruminococcus_gnavus* | 3β-ursodeoxycholic acid | 0.327 | 1.09E-02 | 3.88E-02 |
| *Ruminococcus_gnavus* | 7-ketolithocholic acid | 0.359 | 4.90E-03 | 1.96E-02 |
| *Akkermansia* | Propionic acid | -0.063 | 6.35E-01 | 7.41E-01 |
| *Akkermansia* | Butyric acid | 0.120 | 3.61E-01 | 5.48E-01 |
| *Akkermansia* | Acetic acid | -0.162 | 2.17E-01 | 4.01E-01 |
| *Akkermansia* | Nor-deoxycholic acid | 0.359 | 4.90E-03 | 1.96E-02 |
| *Akkermansia* | Isoallolithocholic acid | 0.548 | 5.81E-06 | 7.59E-05 |
| *Akkermansia* | α-muricholic acid | 0.431 | 5.80E-04 | 4.37E-03 |
| *Akkermansia* | Cholic acid 7 sulfate | 0.192 | 1.42E-01 | 3.31E-01 |
| *Akkermansia* | β-muricholic acid | 0.410 | 1.16E-03 | 7.18E-03 |
| *Akkermansia* | 5α-cholanic acid-3α-ol | 0.332 | 9.60E-03 | 3.55E-02 |
| *Akkermansia* | Ursodeoxycholic acid | -0.419 | 8.62E-04 | 5.83E-03 |
| *Akkermansia* | Dehydrolithocholic acid | 0.390 | 2.10E-03 | 1.05E-02 |
| *Akkermansia* | Chenodeoxycholic acid | -0.417 | 9.08E-04 | 5.93E-03 |
| *Akkermansia* | 3β-ursodeoxycholic acid | -0.317 | 1.36E-02 | 4.67E-02 |
| *Akkermansia* | 7-ketolithocholic acid | -0.405 | 1.32E-03 | 7.55E-03 |
| *Alistipes* | Propionic acid | -0.098 | 4.55E-01 | 6.39E-01 |
| *Alistipes* | Butyric acid | 0.078 | 5.52E-01 | 6.99E-01 |
| *Alistipes* | Acetic acid | -0.245 | 5.88E-02 | 1.71E-01 |
| *Alistipes* | Nor-deoxycholic acid | 0.594 | 5.54E-07 | 1.21E-05 |
| *Alistipes* | Isoallolithocholic acid | 0.666 | 6.45E-09 | 4.22E-07 |
| *Alistipes* | α-muricholic acid | 0.639 | 3.91E-08 | 1.28E-06 |
| *Alistipes* | Cholic acid 7 sulfate | 0.256 | 4.84E-02 | 1.48E-01 |
| *Alistipes* | β-muricholic acid | 0.654 | 1.43E-08 | 5.60E-07 |
| *Alistipes* | 5α-cholanic acid-3α-ol | 0.433 | 5.57E-04 | 4.37E-03 |
| *Alistipes* | Ursodeoxycholic acid | -0.461 | 2.12E-04 | 1.89E-03 |
| *Alistipes* | Dehydrolithocholic acid | 0.581 | 1.12E-06 | 1.82E-05 |
| *Alistipes* | Chenodeoxycholic acid | -0.536 | 1.03E-05 | 1.19E-04 |
| *Alistipes* | 3β-ursodeoxycholic acid | -0.334 | 9.06E-03 | 3.42E-02 |
| *Alistipes* | 7-ketolithocholic acid | -0.471 | 1.46E-04 | 1.36E-03 |
| *Enterobacter* | Propionic acid | -0.081 | 5.37E-01 | 6.90E-01 |
| *Enterobacter* | Butyric acid | -0.183 | 1.62E-01 | 3.60E-01 |
| *Enterobacter* | Acetic acid | 0.011 | 9.34E-01 | 9.73E-01 |
| *Enterobacter* | Nor-deoxycholic acid | -0.102 | 4.40E-01 | 6.29E-01 |
| *Enterobacter* | Isoallolithocholic acid | -0.242 | 6.29E-02 | 1.79E-01 |
| *Enterobacter* | α-muricholic acid | -0.181 | 1.67E-01 | 3.60E-01 |
| *Enterobacter* | Cholic acid 7 sulfate | 0.004 | 9.74E-01 | 9.94E-01 |
| *Enterobacter* | β-muricholic acid | -0.159 | 2.25E-01 | 4.06E-01 |
| *Enterobacter* | 5α-cholanic acid-3α-ol | -0.035 | 7.89E-01 | 8.75E-01 |
| *Enterobacter* | Ursodeoxycholic acid | 0.084 | 5.23E-01 | 6.81E-01 |
| *Enterobacter* | Dehydrolithocholic acid | -0.084 | 5.24E-01 | 6.81E-01 |
| *Enterobacter* | Chenodeoxycholic acid | 0.066 | 6.17E-01 | 7.28E-01 |
| *Enterobacter* | 3β-ursodeoxycholic acid | -0.057 | 6.66E-01 | 7.68E-01 |
| *Enterobacter* | 7-ketolithocholic acid | 0.102 | 4.38E-01 | 6.29E-01 |
| *Escherichia* | Propionic acid | 0.129 | 3.25E-01 | 5.01E-01 |
| *Escherichia* | Butyric acid | 0.000 | 9.98E-01 | 1.00E+00 |
| *Escherichia* | Acetic acid | 0.194 | 1.38E-01 | 3.31E-01 |
| *Escherichia* | Nor-deoxycholic acid | -0.114 | 3.86E-01 | 5.82E-01 |
| *Escherichia* | Isoallolithocholic acid | -0.192 | 1.42E-01 | 3.31E-01 |
| *Escherichia* | α-muricholic acid | -0.195 | 1.35E-01 | 3.31E-01 |
| *Escherichia* | Cholic acid 7 sulfate | -0.001 | 9.96E-01 | 1.00E+00 |
| *Escherichia* | β-muricholic acid | -0.203 | 1.21E-01 | 3.11E-01 |
| *Escherichia* | 5α-cholanic acid-3α-ol | -0.066 | 6.15E-01 | 7.28E-01 |
| *Escherichia* | Ursodeoxycholic acid | 0.182 | 1.64E-01 | 3.60E-01 |
| *Escherichia* | Dehydrolithocholic acid | -0.111 | 4.00E-01 | 5.98E-01 |
| *Escherichia* | Chenodeoxycholic acid | 0.146 | 2.65E-01 | 4.44E-01 |
| *Escherichia* | 3β-ursodeoxycholic acid | 0.140 | 2.85E-01 | 4.61E-01 |
| *Escherichia* | 7-ketolithocholic acid | 0.191 | 1.44E-01 | 3.31E-01 |
| *Oscillibacter* | Propionic acid | -0.152 | 2.45E-01 | 4.17E-01 |
| *Oscillibacter* | Butyric acid | 0.143 | 2.76E-01 | 4.51E-01 |
| *Oscillibacter* | Acetic acid | -0.234 | 7.19E-02 | 1.98E-01 |
| *Oscillibacter* | Nor-deoxycholic acid | 0.600 | 4.08E-07 | 9.99E-06 |
| *Oscillibacter* | Isoallolithocholic acid | 0.719 | 1.02E-10 | 1.00E-08 |
| *Oscillibacter* | α-muricholic acid | 0.654 | 1.43E-08 | 5.60E-07 |
| *Oscillibacter* | Cholic acid 7 sulfate | 0.217 | 9.52E-02 | 2.56E-01 |
| *Oscillibacter* | β-muricholic acid | 0.722 | 7.57E-11 | 1.00E-08 |
| *Oscillibacter* | 5α-cholanic acid-3α-ol | 0.592 | 6.42E-07 | 1.26E-05 |
| *Oscillibacter* | Ursodeoxycholic acid | -0.584 | 9.64E-07 | 1.72E-05 |
| *Oscillibacter* | Dehydrolithocholic acid | 0.603 | 3.46E-07 | 9.69E-06 |
| *Oscillibacter* | Chenodeoxycholic acid | -0.577 | 1.37E-06 | 2.06E-05 |
| *Oscillibacter* | 3β-ursodeoxycholic acid | -0.409 | 1.17E-03 | 7.18E-03 |
| *Oscillibacter* | 7-ketolithocholic acid | -0.558 | 3.54E-06 | 4.95E-05 |
| *Roseburia* | Propionic acid | 0.256 | 4.83E-02 | 1.48E-01 |
| *Roseburia* | Butyric acid | 0.543 | 7.34E-06 | 9.00E-05 |
| *Roseburia* | Acetic acid | 0.348 | 6.38E-03 | 2.45E-02 |
| *Roseburia* | Nor-deoxycholic acid | 0.067 | 6.12E-01 | 7.28E-01 |
| *Roseburia* | Isoallolithocholic acid | 0.043 | 7.47E-01 | 8.37E-01 |
| *Roseburia* | α-muricholic acid | -0.013 | 9.23E-01 | 9.72E-01 |
| *Roseburia* | Cholic acid 7 sulfate | -0.074 | 5.73E-01 | 7.06E-01 |
| *Roseburia* | β-muricholic acid | -0.016 | 9.06E-01 | 9.65E-01 |
| *Roseburia* | 5α-cholanic acid-3α-ol | -0.035 | 7.91E-01 | 8.75E-01 |
| *Roseburia* | Ursodeoxycholic acid | 0.062 | 6.35E-01 | 7.41E-01 |
| *Roseburia* | Dehydrolithocholic acid | 0.028 | 8.32E-01 | 9.06E-01 |
| *Roseburia* | Chenodeoxycholic acid | 0.105 | 4.24E-01 | 6.27E-01 |
| *Roseburia* | 3β-ursodeoxycholic acid | 0.077 | 5.59E-01 | 6.99E-01 |
| *Roseburia* | 7-ketolithocholic acid | 0.000 | 1.00E+00 | 1.00E+00 |
| *Ruthenibacterium* | Propionic acid | -0.187 | 1.52E-01 | 3.46E-01 |
| *Ruthenibacterium* | Butyric acid | -0.077 | 5.57E-01 | 6.99E-01 |
| *Ruthenibacterium* | Acetic acid | -0.371 | 3.53E-03 | 1.54E-02 |
| *Ruthenibacterium* | Nor-deoxycholic acid | 0.381 | 2.69E-03 | 1.20E-02 |
| *Ruthenibacterium* | Isoallolithocholic acid | 0.435 | 5.09E-04 | 4.16E-03 |
| *Ruthenibacterium* | α-muricholic acid | 0.382 | 2.63E-03 | 1.20E-02 |
| *Ruthenibacterium* | Cholic acid 7 sulfate | 0.383 | 2.54E-03 | 1.18E-02 |
| *Ruthenibacterium* | β-muricholic acid | 0.407 | 1.25E-03 | 7.44E-03 |
| *Ruthenibacterium* | 5α-cholanic acid-3α-ol | 0.493 | 6.25E-05 | 6.80E-04 |
| *Ruthenibacterium* | Ursodeoxycholic acid | -0.289 | 2.51E-02 | 8.21E-02 |
| *Ruthenibacterium* | Dehydrolithocholic acid | 0.474 | 1.32E-04 | 1.30E-03 |
| *Ruthenibacterium* | Chenodeoxycholic acid | -0.402 | 1.43E-03 | 7.81E-03 |
| *Ruthenibacterium* | 3β-ursodeoxycholic acid | -0.177 | 1.77E-01 | 3.68E-01 |
| *Ruthenibacterium* | 7-ketolithocholic acid | -0.276 | 3.28E-02 | 1.04E-01 |

**Supplementary Table S4.** The correlations between differential microbiota,metabolites and clinical parameters.

| microbiota/metabolites | clinical parameters | r-value | *P*-value | FDR-value |
| --- | --- | --- | --- | --- |
| Propionic acid | BSFS | 0.346 | 6.85E-03 | 2.74E-02 |
| Butyric acid | BSFS | 0.285 | 2.74E-02 | 5.68E-02 |
| Acetic acid | BSFS | 0.520 | 2.05E-05 | 3.82E-04 |
| Nor-deoxycholic acid | BSFS | -0.220 | 9.13E-02 | 1.46E-01 |
| Isoallolithocholic acid | BSFS | -0.365 | 4.17E-03 | 2.12E-02 |
| α-muricholic acid | BSFS | -0.402 | 1.47E-03 | 1.03E-02 |
| cholic acid 7 sulfate | BSFS | -0.170 | 1.93E-01 | 2.39E-01 |
| β-muricholic acid | BSFS | -0.417 | 9.16E-04 | 7.46E-03 |
| 5α-cholanic acid-3α-ol | BSFS | -0.290 | 2.46E-02 | 5.31E-02 |
| Ursodeoxycholic acid | BSFS | 0.335 | 8.93E-03 | 3.33E-02 |
| Dehydrolithocholic acid | BSFS | -0.311 | 1.55E-02 | 4.83E-02 |
| Chenodeoxycholic acid | BSFS | 0.354 | 5.53E-03 | 2.38E-02 |
| 3β-ursodeoxycholic acid | BSFS | 0.305 | 1.80E-02 | 5.03E-02 |
| 7-ketolithocholic acid | BSFS | 0.298 | 2.07E-02 | 5.14E-02 |
| *Alistipes_onderdonkii* | BSFS | -0.292 | 2.38E-02 | 5.31E-02 |
| *Enterobacter_cloacae_complex* | BSFS | 0.236 | 6.97E-02 | 1.26E-01 |
| *Escherichia_coli* | BSFS | 0.276 | 3.30E-02 | 6.34E-02 |
| *Klebsiella_pneumoniae* | BSFS | 0.216 | 9.71E-02 | 1.47E-01 |
| *Megamonas_funiformis* | BSFS | 0.097 | 4.62E-01 | 4.80E-01 |
| *Ruthenibacterium_lactatiformans* | BSFS | -0.224 | 8.53E-02 | 1.40E-01 |
| *Ruminococcus_gnavus* | BSFS | 0.104 | 4.29E-01 | 4.53E-01 |
| *Akkermansia* | BSFS | -0.175 | 1.82E-01 | 2.36E-01 |
| *Alistipes* | BSFS | -0.227 | 8.05E-02 | 1.37E-01 |
| *Enterobacter* | BSFS | -0.028 | 8.30E-01 | 8.45E-01 |
| *Escherichia* | BSFS | 0.132 | 3.14E-01 | 3.52E-01 |
| *Oscillibacter* | BSFS | -0.313 | 1.49E-02 | 4.83E-02 |
| *Roseburia* | BSFS | 0.302 | 1.92E-02 | 5.11E-02 |
| *Ruthenibacterium* | BSFS | -0.229 | 7.87E-02 | 1.37E-01 |
| Propionic acid | SBMs | 0.546 | 6.33E-06 | 1.77E-04 |
| Butyric acid | SBMs | 0.376 | 3.05E-03 | 1.71E-02 |
| Acetic acid | SBMs | 0.579 | 1.24E-06 | 6.97E-05 |
| Nor-deoxycholic acid | SBMs | -0.172 | 1.90E-01 | 2.39E-01 |
| Isoallolithocholic acid | SBMs | -0.305 | 1.77E-02 | 5.03E-02 |
| α-muricholic acid | SBMs | -0.297 | 2.12E-02 | 5.14E-02 |
| cholic acid 7 sulfate | SBMs | -0.184 | 1.60E-01 | 2.13E-01 |
| β-muricholic acid | SBMs | -0.325 | 1.12E-02 | 3.91E-02 |
| 5α-cholanic acid-3α-ol | SBMs | -0.166 | 2.04E-01 | 2.39E-01 |
| Ursodeoxycholic acid | SBMs | 0.420 | 8.41E-04 | 7.46E-03 |
| Dehydrolithocholic acid | SBMs | -0.142 | 2.80E-01 | 3.20E-01 |
| Chenodeoxycholic acid | SBMs | 0.417 | 9.32E-04 | 7.46E-03 |
| 3β-ursodeoxycholic acid | SBMs | 0.489 | 7.34E-05 | 1.03E-03 |
| 7-ketolithocholic acid | SBMs | 0.396 | 1.76E-03 | 1.09E-02 |
| *Alistipes_onderdonkii* | SBMs | -0.214 | 1.00E-01 | 1.48E-01 |
| *Enterobacter_cloacae_complex* | SBMs | 0.166 | 2.05E-01 | 2.39E-01 |
| *Escherichia_coli* | SBMs | 0.116 | 3.77E-01 | 4.14E-01 |
| *Klebsiella_pneumoniae* | SBMs | 0.168 | 1.99E-01 | 2.39E-01 |
| *Megamonas_funiformis* | SBMs | 0.218 | 9.43E-02 | 1.47E-01 |
| *Ruthenibacterium_lactatiformans* | SBMs | -0.187 | 1.52E-01 | 2.08E-01 |
| *Ruminococcus_gnavus* | SBMs | 0.110 | 4.03E-01 | 4.34E-01 |
| *Akkermansia* | SBMs | -0.283 | 2.87E-02 | 5.73E-02 |
| *Alistipes* | SBMs | -0.274 | 3.40E-02 | 6.34E-02 |
| *Enterobacter* | SBMs | 0.019 | 8.84E-01 | 8.84E-01 |
| *Escherichia* | SBMs | 0.203 | 1.20E-01 | 1.73E-01 |
| *Oscillibacter* | SBMs | -0.356 | 5.19E-03 | 2.38E-02 |
| *Roseburia* | SBMs | 0.197 | 1.32E-01 | 1.85E-01 |
| *Ruthenibacterium* | SBMs | -0.295 | 2.20E-02 | 5.14E-02 |
| Propionic acid | CSS | -0.144 | 3.75E-01 | 7.92E-01 |
| Butyric acid | CSS | -0.199 | 2.18E-01 | 7.63E-01 |
| Acetic acid | CSS | -0.249 | 1.21E-01 | 6.76E-01 |
| Nor-deoxycholic acid | CSS | -0.017 | 9.15E-01 | 9.49E-01 |
| Isoallolithocholic acid | CSS | 0.071 | 6.64E-01 | 7.94E-01 |
| α-muricholic acid | CSS | 0.089 | 5.84E-01 | 7.92E-01 |
| cholic acid 7 sulfate | CSS | 0.083 | 6.10E-01 | 7.92E-01 |
| β-muricholic acid | CSS | 0.091 | 5.78E-01 | 7.92E-01 |
| 5α-cholanic acid-3α-ol | CSS | -0.067 | 6.81E-01 | 7.94E-01 |
| Ursodeoxycholic acid | CSS | -0.301 | 5.92E-02 | 6.76E-01 |
| Dehydrolithocholic acid | CSS | 0.004 | 9.82E-01 | 9.82E-01 |
| Chenodeoxycholic acid | CSS | -0.109 | 5.01E-01 | 7.92E-01 |
| 3β-ursodeoxycholic acid | CSS | -0.222 | 1.69E-01 | 6.76E-01 |
| 7-ketolithocholic acid | CSS | -0.085 | 6.02E-01 | 7.92E-01 |
| *Alistipes_onderdonkii* | CSS | -0.023 | 8.87E-01 | 9.49E-01 |
| *Enterobacter_cloacae_complex* | CSS | -0.140 | 3.87E-01 | 7.92E-01 |
| *Escherichia_coli* | CSS | 0.050 | 7.61E-01 | 8.53E-01 |
| *Klebsiella_pneumoniae* | CSS | -0.137 | 3.99E-01 | 7.92E-01 |
| *Megamonas_funiformis* | CSS | -0.281 | 7.89E-02 | 6.76E-01 |
| *Ruthenibacterium_lactatiformans* | CSS | 0.230 | 1.53E-01 | 6.76E-01 |
| *Ruminococcus_gnavus* | CSS | -0.107 | 5.12E-01 | 7.92E-01 |
| *Akkermansia* | CSS | 0.080 | 6.22E-01 | 7.92E-01 |
| *Alistipes* | CSS | 0.133 | 4.14E-01 | 7.92E-01 |
| *Enterobacter* | CSS | -0.086 | 5.99E-01 | 7.92E-01 |
| *Escherichia* | CSS | -0.186 | 2.52E-01 | 7.83E-01 |
| *Oscillibacter* | CSS | 0.097 | 5.50E-01 | 7.92E-01 |
| *Roseburia* | CSS | -0.337 | 3.37E-02 | 6.76E-01 |
| *Ruthenibacterium* | CSS | 0.222 | 1.69E-01 | 6.76E-01 |

## Supplementary Figures

| 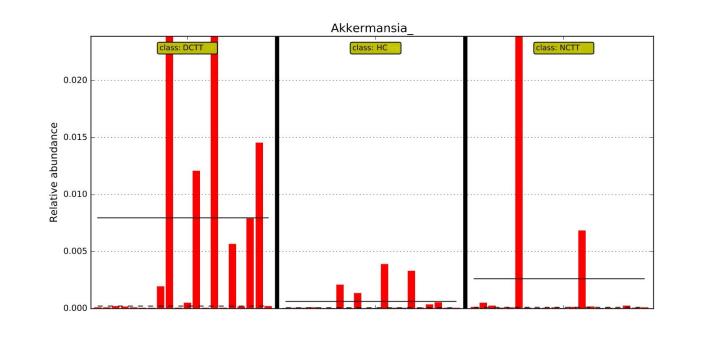 | 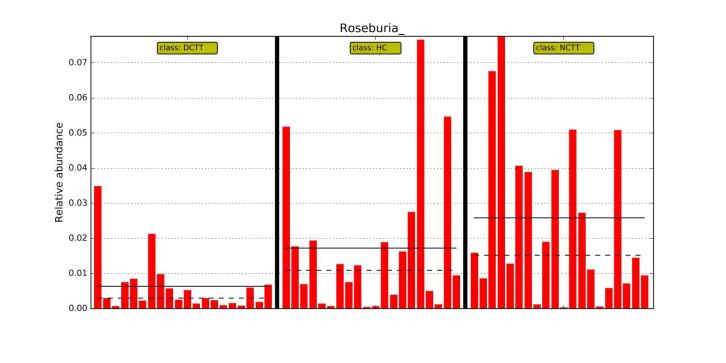 |
| --- | --- |
| 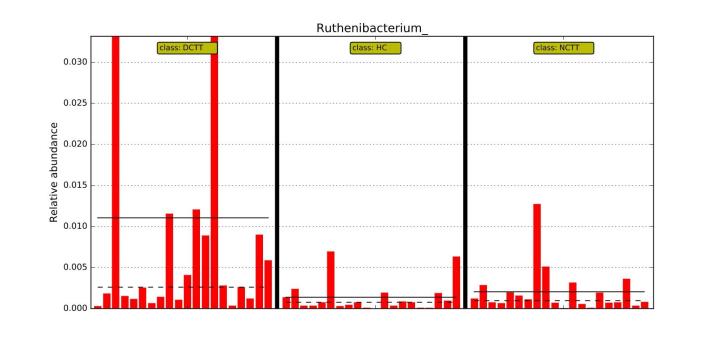 | 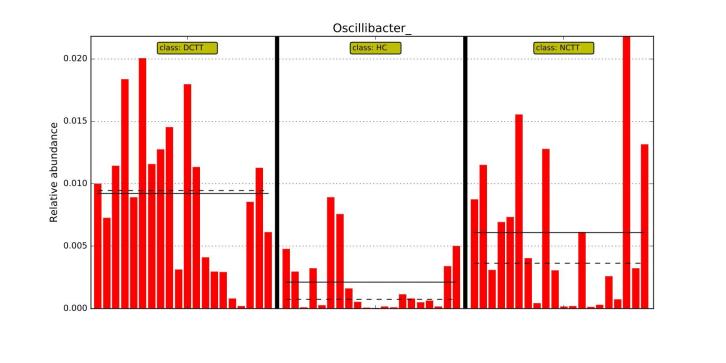 |
| 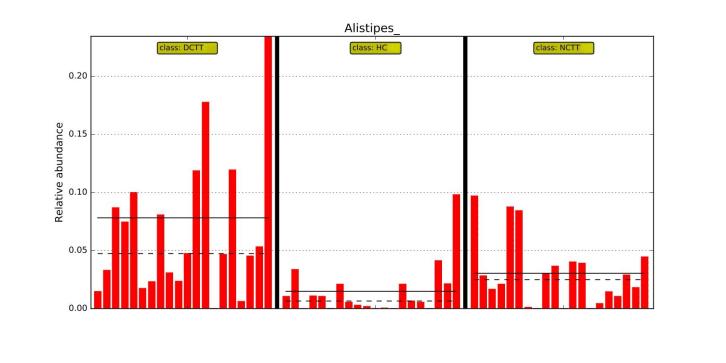 | 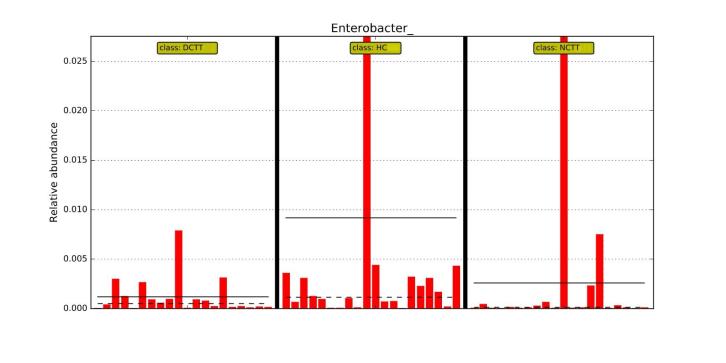 |
| 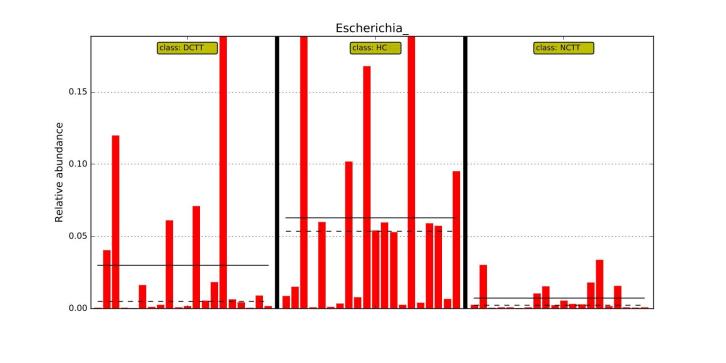 | 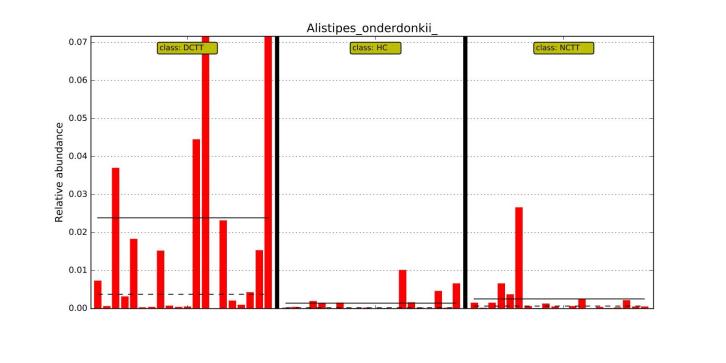 |
| 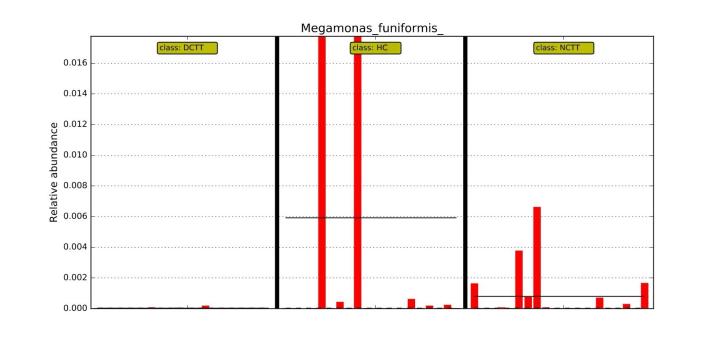 | 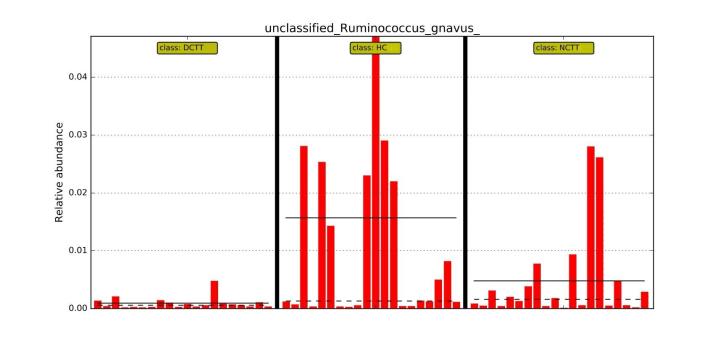 |
| 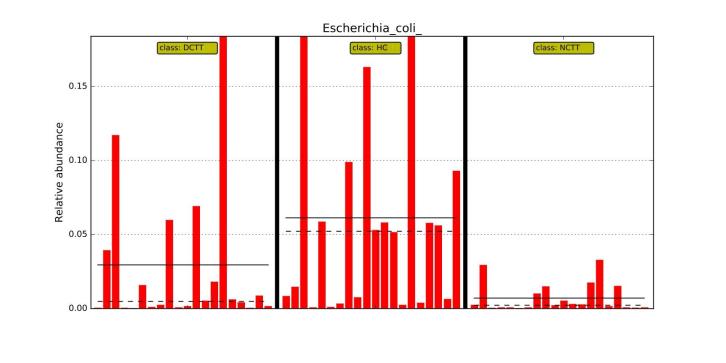 | 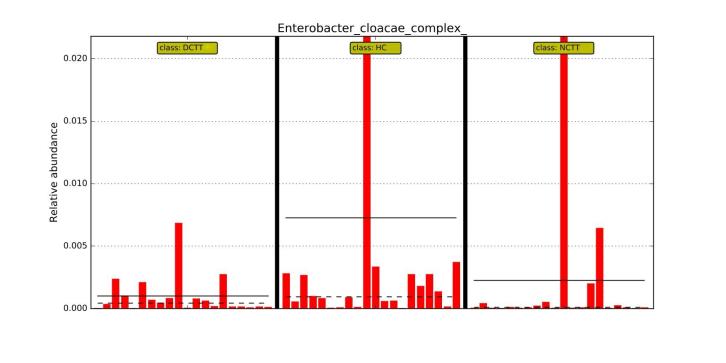 |
| 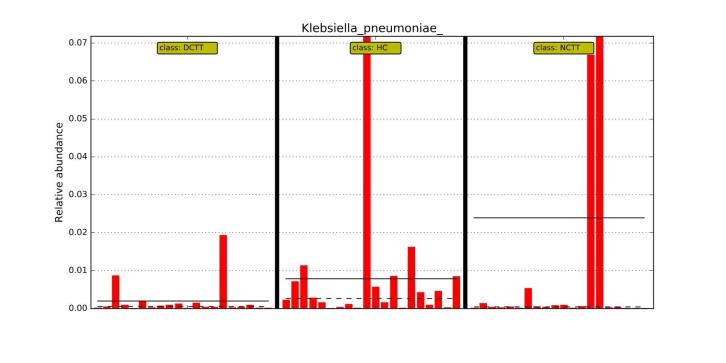 | 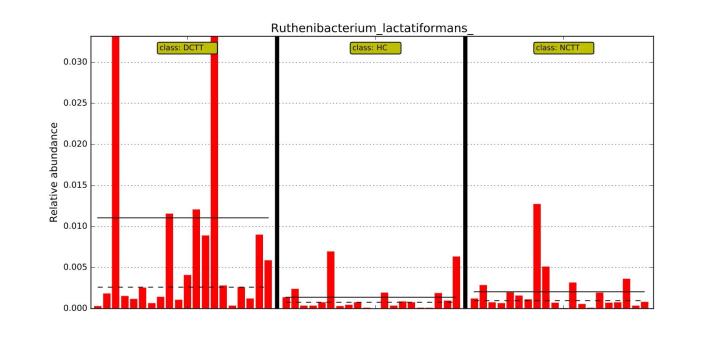 |

**Supplementary Figure 1.** Relative abundance levels of 14 keystone bacteria in each sample.
